# Assembly of functional photosystem complexes in *Rhodobacter sphaeroides* incorporating carotenoids from the spirilloxanthin pathway

**DOI:** 10.1016/j.bbabio.2014.10.004

**Published:** 2015-02

**Authors:** Shuang C. Chi, David J. Mothersole, Preston Dilbeck, Dariusz M. Niedzwiedzki, Hao Zhang, Pu Qian, Cvetelin Vasilev, Katie J. Grayson, Philip J. Jackson, Elizabeth C. Martin, Ying Li, Dewey Holten, C. Neil Hunter

**Affiliations:** a Department of Molecular Biology and Biotechnology, University of Sheffield, Sheffield S10 2TN, United Kingdom; b Department of Chemistry, Washington University, St. Louis, MO 63130-4889, USA; c ChELSI Institute, Department of Chemical and Biological Engineering, University of Sheffield, Mappin Street, Sheffield S1 3JD, United Kingdom; d Department of Microbiology and Immunology, China Agricultural University, Haidian District, Beijing 100193, China; e Photosynthetic Antenna Research Center, Washington University, St. Louis, MO 63130 USA

**Keywords:** BChl(s), bacteriochlorophyll(s), B850, bacteriochlorophyll in light harvesting 2 complex with absorption maximum at 850 nm, B875, bacteriochlorophyll in light harvesting 1 complex with absorption maximum at 875 nm, EDTA, ethylenediamine tetraacetic acid, HEPES, N-2-hydroxyethylpiperazine-N′-2-ethanesulfonic acid, ICM, intracytoplasmic membrane, LH1, light-harvesting 1 complex, LH2, light-harvesting 2 complex, MS, mass spectrometry, *P.*, *Pantoea*, *Phs.*, *Phaeospirillum*, *Rba.*, *Rhodobacter*, RC, reaction centre, RC-LH1, reaction centre-light-harvesting 1 complex, *Rsp.*, *Rhodospirillum*, *Rps.*, *Rhodopseudomonas*, β-DDM, β-dodecylmaltoglucoside, OD, optical density, WT, wild type, Bacterial photosynthesis, Light harvesting, Carotenoid, Membrane protein, Antenna, Synthetic biology

## Abstract

Carotenoids protect the photosynthetic apparatus against harmful radicals arising from the presence of both light and oxygen. They also act as accessory pigments for harvesting solar energy, and are required for stable assembly of many light-harvesting complexes. In the phototrophic bacterium *Rhodobacter* (*Rba.*) *sphaeroides* phytoene desaturase (CrtI) catalyses three sequential desaturations of the colourless carotenoid phytoene, extending the number of conjugated carbon–carbon double bonds, *N*, from three to nine and producing the yellow carotenoid neurosporene; subsequent modifications produce the yellow/red carotenoids spheroidene/spheroidenone (*N* = 10/11). Genomic *crtI* replacements were used to swap the native three-step *Rba. sphaeroides* CrtI for the four-step *Pantoea agglomerans* enzyme, which re-routed carotenoid biosynthesis and culminated in the production of 2,2′-diketo-spirilloxanthin under semi-aerobic conditions. The new carotenoid pathway was elucidated using a combination of HPLC and mass spectrometry. Premature termination of this new pathway by inactivating *crtC* or *crtD* produced strains with lycopene or rhodopin as major carotenoids. All of the spirilloxanthin series carotenoids are accepted by the assembly pathways for LH2 and RC–LH1–PufX complexes. The efficiency of carotenoid-to-bacteriochlorophyll energy transfer for 2,2′-diketo-spirilloxanthin (15 conjugated C=C bonds; *N* = 15) in LH2 complexes is low, at 35%. High energy transfer efficiencies were obtained for neurosporene (*N* = 9; 94%), spheroidene (*N* = 10; 96%) and spheroidenone (*N* = 11; 95%), whereas intermediate values were measured for lycopene (*N* = 11; 64%), rhodopin (*N* = 11; 62%) and spirilloxanthin (*N* = 13; 39%). The variety and stability of these novel *Rba. sphaeroides* antenna complexes make them useful experimental models for investigating the energy transfer dynamics of carotenoids in bacterial photosynthesis.

## Highlights

• The spirilloxanthin biosynthetic pathway has been constructed in *Rba. sphaeroides*.
• The new carotenoids are accepted by the photosystem assembly pathways.
• These pigments are efficiently integrated into LH2 and RC–LH1–PufX complexes.
• Carotenoid–BChl energy transfer drops with the number of conjugated C=C bonds (*N*).
• The lowest efficiency, 35%, is for the *N* = 15 carotenoid 2,2′ diketospirilloxanthin.

## Graphical abstract

**Figure:**
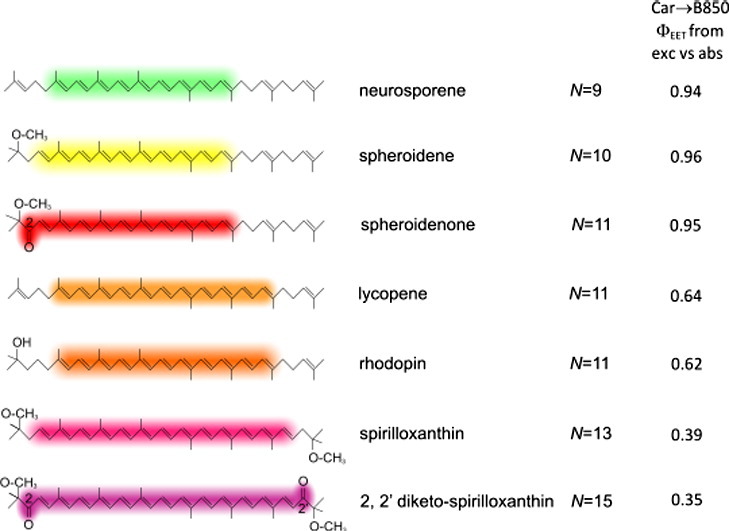

## 1 Introduction

The carotenoids spheroidenone and spheroidene in the photosynthetic bacterium *Rhodobacter* (*Rba.*) *sphaeroides* assist in light harvesting, prevent the formation of singlet state oxygen through quenching chlorophyll triplet states and act as stabilising structural components for the light-harvesting LH2 complex. Solar energy absorbed by the carotenoids and bacteriochlorophylls (BChls) within LH2 passes to the LH1 complex, which surrounds and interconnects reaction centres (RCs), the complexes that convert excitation energy to a charge separation. Arrays of LH2 and RC-LH1 complexes form a light-harvesting and energy trapping network embedded in intracytoplasmic membranes (ICM) [1]. The complex ICM network of *Rba. sphaeroides* comprises hundreds of ICM vesicles, found as separate spherical membranes and as interconnected structures providing a large surface area for harvesting and utilising solar energy [2], [3].

Carotenoids are essential for the stable assembly of the *Rba. sphaeroides* LH2 complex [4], [5], although not for the *Chromatium minutissimum* LH2 complex [6]. The structure of the *Rhodopseudomonas (Rps.) acidophila* LH2 complex shows the basis for this structural role; nine membrane-spanning carotenoids traverse the membrane, making close contacts with both the nine B800 and eighteen B850 BChls [7]. The B850 BChls are bound to the α and β transmembrane polypeptides forming an eighteen-membered ring of overlapping pigments. A similar, although larger LH ring is found for the LH1 antenna that encloses the RC, although this LH complex requires no carotenoids for its assembly [5], [8]. A recent spectroscopic study did however establish a clear functional role for carotenoids in the *Rba. sphaeroides* LH1 complex; ultrafast transient absorption spectroscopy showed that spheroidenone is an effective quencher of singlet oxygen, whereas LH1 complexes lose their photoprotection in the presence of spheroidene [9].

**Fig. 1:**
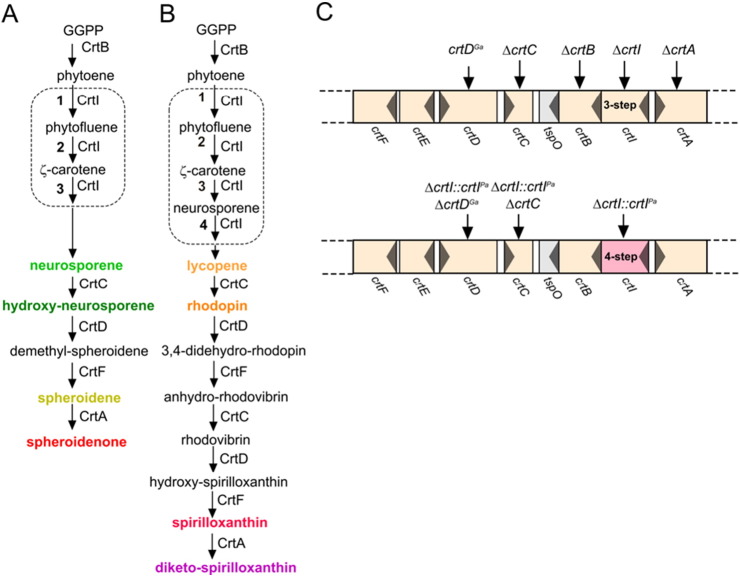
Carotenoid biosynthesis pathways and genes. (A) The spheroidene/spheroidenone pathway typical of *Rba. sphaeroides*. The three reactions catalysed by phytoene desaturase (CrtI) are shown within the dashed outline. (B) The spirilloxanthin pathway, as shown in [10]. The four reactions catalysed by phytoene desaturase (CrtI) are shown within the dashed outline. The text labelling a particular carotenoid corresponds to the colour of the corresponding carotenoid mutant. (C) Genetic manipulation of the *Rba. sphaeroides* carotenoid biosynthetic pathway. The upper cluster of native *crt* genes is shown, plus the gene alterations used in this study. In the lower cluster the native *crtI* gene has been replaced with the *P. agglomerans* gene encoding the four-step phytoene desaturase, indicated by Δ*crtI::crtI^Pa^*. Following this replacement, other gene modifications were carried out as indicated.

Carotenoids are synthesised by the sequential desaturation of the colourless pigment phytoene, which has three conjugated double bonds [10]. In *Rba. sphaeroides* phytoene desaturase produces the yellow pigment neurosporene (three desaturations, nine conjugated double bonds: *N* = 9), whereas in many other phototrophic bacteria such as *Rhodospirillum (Rsp.) rubrum*, *Rps. acidophila* and *Phaeospirillum (Phs.) molischianum*, a further desaturation by the four-step phytoene desaturase produces the orange carotenoid lycopene (four desaturations, *N* = 11). Subsequently, a series of modifications introduces double bonds, hydroxy and methoxy groups, with the end products being spheroidene/spheroidenone in the case of *Rhodobacter* or spirilloxanthin in the case of *Rhodospirillum (Rsp.) rubrum*. Sometimes the pathway terminates prematurely; for example, *Rps. acidophila* uses the spirilloxanthin pathway but synthesises rhodopin and rhodopin glucoside (see Fig. 1). The only difference between these bacteria appears to be the possession of a three- or four step phytoene desaturase; this was shown by an early carotenoid engineering study that swapped the native three-step phytoene desaturase for the four-step enzyme from *Erwinia herbicola* (now *Pantoea (P.) agglomerans*); further processing of lycopene had been prevented by blocking the pathway at the level of CrtC, so lycopene became the major carotenoid in *Rba. sphaeroides* [11]. It was found that lycopene entered the LH2 assembly pathway and, once incorporated into the new complex, this pigment transfers absorbed light energy to the BChls with an efficiency that compares favourably with other LH2 complexes that contain *N* = 11 carotenoids [11], [12], [13], [14]. Thus, the type of phytoene desaturase appears to determine the carotenoid pathway that is followed in a particular organism [15], but the level of desaturation appears to have no bearing on the activity of subsequent pathway enzymes.

The availability of the pK18mobsacB plasmid [16] for constructing serial gene deletions, insertions and swaps allows extensive reconfiguration of bacterial genomes. In particular we can now customise the carotenoid pathway to produce a series of non-native carotenoids, and examine the consequences for assembly and energy transfer in LH2 and RC-LH1 complexes. In the present work we have explored the consequence of genomic swapping of the native three-step phytoene desaturase for the four-step *P. agglomerans* enzyme, creating a new ‘full-length’ pathway in *Rba. sphaeroides* that produces 2,2′-diketo-spirilloxanthin under semi-aerobic conditions. This three/four-step phytoene desaturase swap was combined with a series of genomically-encoded premature terminations of the carotenoid pathway that yield lycopene, rhodopin or spirilloxanthin. The extent to which these new carotenoids are tolerated by the assembly machinery for LH2, RC–LH1–PufX monomer and RC–LH1–PufX dimer complexes has been investigated and their function, in terms of energy transfer to BChls, has been quantified. In general, all of the non-native carotenoids are accepted by these complexes, and their energy transfer properties change in a graded way as the number of conjugated double bonds increases, as found with *in vitro* reconstitutions [13], [17], [18], [19]. There are also some surprising outcomes, with some non-native carotenoids appearing to promote abnormally high levels of RC–LH1–PufX dimer formation.

## 2 Materials and methods

### 2.1 *Escherichia coli* strains and plasmids

Two strains of *E. coli* were primarily used in this work; JM109 chemically competent cells purchased from Sigma, and S17-1 [20]. S17-1 cells were used for plasmid transfer into *Rba. sphaeroides* strains and were made electrocompetent. Strains were grown in Luria-Bertani (LB) medium with antibiotics added when required. The following antibiotic concentrations (μg ml^− 1^) were used: kanamycin 30; ampicillin 200; and tetracycline, 10.

### 2.2 *Rba. sphaeroides* strains

The wild-type *Rba. sphaeroides* strain 2.4.1 and subsequent mutants were grown in M22 + medium [21]; 0.1% casamino acids were used to supplement liquid cultures. Stocks of strains were stored at − 80 °C in LB medium containing 50% glycerol (v/v).

### 2.3 Construction of new carotenoid biosynthesis clusters: conjugative transfer of plasmid DNA from *E. coli* to *Rba. sphaeroides* and selection of *Rba. sphaeroides* mutants on sucrose

The pK18mob*sacB* [16] plasmids containing the *crt* gene constructs destined for integration into the *Rba. sphaeroides* genome were transferred by conjugation from *E. coli* strain S17-1 to the desired *Rba. sphaeroides* strain. Single kanamycin-resistant transconjugant colonies were grown and scaled up to an 80 ml semi aerobic culture. The cells were serially diluted on M22+ agar containing 10% (w/v) sucrose and incubated for 4–6 days until single colonies appeared. Single colonies were replica-plated on M22 + sucrose containing no antibiotic selection and M22+/sucrose/kanamycin. Colonies for deletion mutants that grew on the antibiotic-free plate but not on the kanamycin containing plate were analysed by PCR. Colonies were checked for insertion of the *crtI* encoding the four-step phytoene desaturase by DNA sequencing.

### 2.4 Photosynthetic and semiaerobic growth of *Rba. sphaeroides* strains

Anaerobic cultures of *Rba. sphaeroides* were grown under photosynthetic conditions using 15 W or 20 W MEGAMAN® CFL bulbs. The light intensity was 150 μmol photons s^− 1^ m^− 1^, measured using a LI-250A Light Meter equipped with a LI-190 Quantum Sensor (LI-COR Biosciences). 1 ml of semi-aerobic culture was used to inoculate a full 30 ml universal of M22+ medium. The culture was incubated overnight at the desired light intensity, with gentle agitation by a small magnetic stir bar. This culture was used to inoculate a 500 ml medical flat bottle filled with M22+ medium and capped with a rubber bung. These cultures also contained a magnetic stir bar to allow for gentle agitation. Semi-aerobic growth conditions used 2 l flasks, filled to 75% capacity and shaking in the dark at 180 rpm at 30 °C.

### 2.5 Cell harvesting and breakage, and preparation of intracytoplasmic membranes

Cells were pelleted by centrifugation at 4000×*g* at 4 °C for 25–35 min. Cells harvested for absorption spectroscopy, fluorescence spectroscopy or fractionation of the LH2, core complex monomer and dimer bands were resuspended in 20 mM HEPES 5 mM EDTA, pH 7.5. Approximately 5 g of cells was used per 10 ml of buffer. Cells were disrupted by two passages through a French pressure cell at 18,000 psi, and unbroken cells were removed by centrifugation at 33,000×*g* at 4 °C for 25 min. 1 –5 ml of broken cells was layered on top of the 15/40% (w/w) discontinuous sucrose gradient 15% sucrose band using either a peristaltic pump or pipette. Gradients were centrifuged at 27,000 rpm (60,000–65,000×*g*) in a Beckman Type 50.2 Ti or Type 45 Ti rotor at 4 °C for 10 h. A pigmented band of ICM formed at the 15/40% interface and was collected using a fixed needle and a peristaltic pump, then stored at − 20 °C until required.

### 2.6 Fractionation of LH2, core complex monomers and dimers present in ICM membranes

Membranes harvested from discontinuous sucrose gradients were diluted in 20 mM HEPES, pH 7.5 and pelleted at 45,000 rpm (180,000 ×*g*) for 2.5 h using a Beckman Type 50.2 Ti rotor at 4 °C. Pelleted membranes were resuspended in approximately 100–200 μl of 20 mM HEPES, pH 7.5 and the absorbance spectrum used to record the OD_875_ value. 7.5 OD_875_ units of resuspended membranes, equivalent to ~ 43 nmol of BChl, were solubilised in 3% β-dodecyl maltoside (β-DDM) in a total volume of 250 μl before 1 h of centrifugation at 15,000 rpm at 4 °C in a refrigerated microcentrifuge. The supernatant was collected and layered on top of a discontinuous sucrose gradient containing 20%, 21.3%, 22.5%, 23.8% and 25% sucrose, 20 mM HEPES and 0.03% β-DDM. Gradients were centrifuged in a Beckman SW41 Ti rotor at 27,000 rpm (90,000 ×*g*) for 40 h. Digital photos of the gradients were taken and pigmented bands were harvested for downstream processing. The dimer:monomer ratios were estimated using ImageJ software (NIH, Bethesda, USA) to analyse densitometry of photographs of the gradients, as described in [22].

### 2.7 Quantification of BChls and carotenoids in LH2 complexes

All steps were carried out under dim light. 100 μl of each LH2 antenna sample was extracted three times using 7:2 acetone:methanol and the extracts were pooled. Carotenoids were partitioned into hexane using three successive extractions of the acetone:methanol layer, and the hexane extracts were pooled, then dried in the dark under a vacuum, then dissolved in a solvent for which an extinction coefficient has already been reported. The millimolar or mM surely extinction coefficients (1 cm path length) used were: hydroxy-neurosporene 149.4 (438 nm, hexane); neurosporene 159.4 (438 nm, hexane) [23]; spheroidene 173.6 (454 nm, hexane) [19]; spheroidenone 122 (482 nm, 7:2 acetone:methanol) [24]; lycopene 182 (476 nm, hexane) [23]; rhodopin 181.5 (473 nm, hexane) [19]; spirilloxanthin 151 (493 nm, hexane) [19]; and 2,2′-diketo-spirilloxanthin 117 (approximated using the data on 2-keto-spirilloxanthin; 518 nm, petroleum ether [25]). For BChl, the acetone:methanol layer was dried in the dark under nitrogen, then redissolved in methanol, and this pigment was quantified using an extinction coefficient at 771 nm of 54.8 mM^− 1^ cm^− 1^ [26].

### 2.8 Extraction and HPLC analysis of carotenoids

The carotenoid extraction was performed in two steps. First, the concentrated LH2 complex sample was dissolved in a mixture of spectroscopic grade methanol and acetone (Sigma) in 1:1 volume ratio. The extract was spun down using bench microcentrifuge and the supernatant was collected. In the second step the remaining pellet was dissolved in tetrahydrofuran (THF) (Sigma) and centrifuged again. The supernatant was collected and mixed with the previous one; this step was repeated until the supernatant became colourless. The final extract was dried under stream of nitrogen, dissolved in HPLC grade acetonitrile (ACN):THF (8:2, v:v) (Sigma) mixture and injected into an Agilent 1100 HPLC system employing a reverse phase Zorbax Eclipse XDB-C18 column (250 mm × 4.6 mm). The HPLC protocol was programmed for a step gradient mobile phase from 100% ACN to (70:30, v:v) ACN:THF (Sigma) within 30 min as follows: (0–5 min, 100–95% ACN, 5–10 min, 96–90% ACN, 10–15 min 90–80% ACN, 15–20 min, 80–75% ACN, 20–30 min, 75–70% ACN). Carotenoids were detected using a 1024-element diode array, with a 190–950 nm wavelength range and a wavelength accuracy of ± 1 nm, self-calibrated with deuterium lines and verified with a holmium oxide filter.

### 2.9 Identification of carotenoids

Pigment samples were analysed by a Bruker Solarix 12T Fourier Transform Ion Cyclotron Resonance (FT-ICR) mass spectrometer coupled with an Atmospheric-Pressure Chemical Ionization (APCI) source (Bruker, Bremen, Germany) with the APCI source set to positive mode. Samples were dissolved HPLC grade methanol (Fisher Scientific) and directly infused into the APCI source with capillary voltage of 2 kV, dry nitrogen flow 4 l/min and temperature 180 °C, nebulizer gas flow 1.0 l/min, and APCI temperature 350 °C. Each spectrum was acquired between m/z 200 and 3000.

### 2.10 Spectroscopy

Absorption spectra of LH2, core monomers and dimer complexes were acquired at room temperature in a 1 cm path-length cuvette on a Cary 50 UV–vis spectrophotometer, with baseline correction as appropriate. For measurements of carotenoid–BChl energy transfer efficiency absorption/absorptance spectra were acquired on a Shimadzu UV-1800 spectrophotometer. Fluorescence studies were carried out on a Horiba Nanolog spectrofluorometer (Edison New Jersey, USA) equipped with a double excitation monochromator with an output slit set to give a band pass of 2 nm. The detection leg consists of an IHR320 imaging spectrograph (input slit width set to give a band pass of 3 nm) and a 100 groove/mm 780 nm blaze grating with a dispersion that gives 0.8 nm per pixel on a 1024 element Synapse CCD array detector. Fluorescence spectra and fluorescence excitation spectra were corrected for the instrument response. Absorption spectra were converted to absorptance (1-transmittance) for comparison with fluorescence excitation spectra and a mild 1/λ^4^ correction applied to remove the effects of light scattering, which affects these spectra primarily at λ < 550 nm. Excitation, emission, and scanning wavelengths are given in the figure legends. LH2 samples for fluorescence and fluorescence excitation studies had a maximum absorbance of ~ 0.15 (1 cm path cuvettes) in the largest absorption features (Soret and Q_y_). All spectra were measured at room temperature.

## 3 Results

### 3.1 Construction of new carotenoid biosynthesis pathways in *Rba. sphaeroides*

Fig. 1 shows the carotenoid biosynthesis pathways typically found in purple phototrophs such as *Rba. sphaeroides*, *Rsp. rubrum*, *Rps. acidophila* and *Phs. molischianum*. In *Rba. sphaeroides* the normal route is initiated by the three-step phytoene desaturase and culminates in the production of spheroidene under anaerobic photosynthetic growth, whereas spheroidenone is the dominant carotenoid in the presence of oxygen, due to the introduction of a C2 keto group catalysed by spheroidene monooxygenase, or CrtA [27], [28], [29], [30], [31].

The native *Rba. sphaeroides crt* gene cluster was modified by performing genomic deletions that cleanly removed either the *crtB*, *I*, *C*, or *A* genes (Fig. 1C). Complete deletion of *crtD* did not yield a stable phenotype, so instead we exploited the known stability of the Ga mutation [32]. The Ga mutation was identified by sequencing *crtD*; a single C → T base change at position 259 alters CAG to a TAG stop codon. The same mutation was introduced into the genome of some of the strains in this study, and is designated as a *crtD^Ga^* mutation to emphasise that it is not a deletion. In another set of manipulations the native *crtI* was replaced with its *P. agglomerans* counterpart, designated as *crtI*^Pa^, which encodes the four-step phytoene desaturase. This seamless replacement retains the native organisation of the *crt* gene cluster, including the native *crtI* promoter [33], [34] and diverts the native pathway towards spirilloxanthin. The newly acquired *crtI*^Pa^ was combined with genomic deletions of *crtC* or *A*, or with the *crtD^Ga^* mutation. In total, five new strains were constructed.

**Fig. 2:**
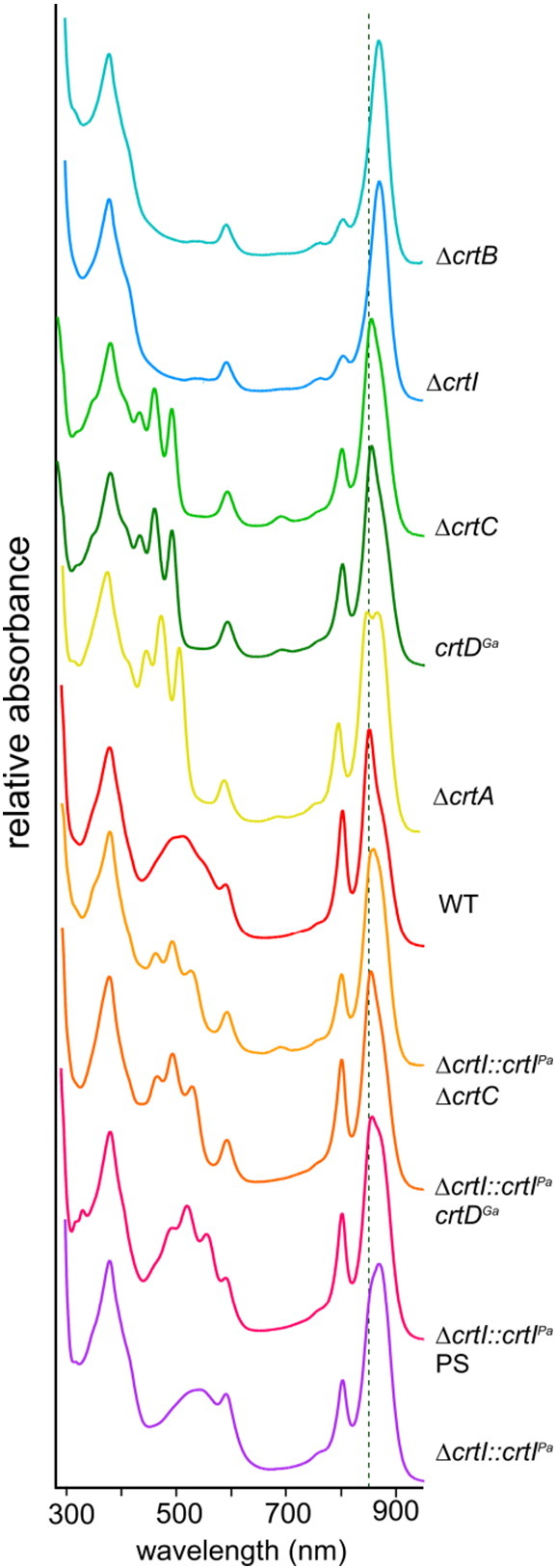
Absorption spectra of ICMs prepared from various carotenoid strains. The dashed line indicating 850 nm is used for comparison of the major near infra-red absorption maxima.

The absorption spectra of membranes prepared from semi-aerobically grown cells (Fig. 2) show appreciable levels of LH2 and RC–LH1–PufX complexes for all carotenoid backgrounds with exception of the Δ*crtB* and Δ*crtI* mutants, which have no LH2 complexes as already reported [5], [27], [22]. In most of these spectra the LH2 complex dominates, but not in the Δ*crtI::crtI^Pa^* semiaerobic membranes containing 2,2′-diketo-spirilloxanthin (Fig. 2, bottom spectrum). To a lesser extent, the same effect is seen for *ΔcrtI::crtI^Pa^*PS (spirilloxanthin), Δ*crtI::crtI^Pa^ crtD^Ga^* (rhodopin) and Δ*crtI::crtI^Pa^* Δ*crtC* (lycopene) samples. These spectra suggest that spirilloxanthin and 2,2′-diketo-spirilloxanthin tend to favour the assembly LH1 complexes rather than LH2.

### 3.2 Fractionation of membranes from new carotenoid strains into LH2 and RC–LH1–PufX complexes

The room temperature spectra in Fig. 2 do not resolve the contributions from LH2 at ~ 850 nm and LH1 at ~ 875 nm, particularly since small absorption shifts arising from the new carotenoids could converge these maxima still further. In order to investigate the effects of these different carotenoids on each photosystem component, detergent-solubilised membranes were fractionated into LH2, monomer RC–LH1–PufX and dimer RC–LH1–PufX complexes. First, the carotenoid strains were grown either anaerobically and photosynthetically or in dark, oxygen-limited heterotrophic cultures. Intracytoplasmic membranes were prepared, solubilised in 3% β-DDM and the extracts were fractionated on discontinuous sucrose density gradients.

**Fig. 3:**
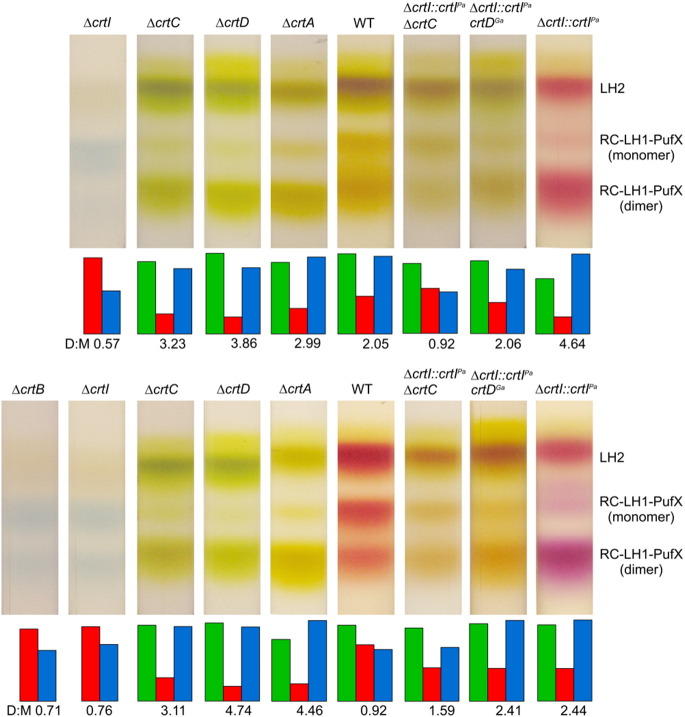
Fractionation of detergent-solubilised membranes on sucrose density gradients. Gradients of solubilised membranes are shown for each carotenoid strain, and from photosynthetically grown (top row) and semi-aerobically grown (bottom row) cells. LH2, and RC –LH1–PufX monomer and dimer bands were quantified using densitometry of the gradient photograph using ImageJ software, and the amounts of each complex are depicted in the histogram underneath each gradient (LH2, green; RC–LH1–PufX monomer, red; RC–LH1–PufX dimer, blue). The number underneath each histogram is the core dimer:monomer ratio calculated for each mutant.

Fig. 3 shows the LH2 (top), monomer RC–LH1–PufX (middle) and dimer RC–LH1–PufX (bottom) bands for each of these samples, prepared from photosynthetically grown cells (upper row), and semiaerobically grown cells (lower row). Fairly consistent distributions of complexes were found for each of these growth conditions. The only exception is the wild-type, where spheroidene monooxygenase (CrtA) converts the yellow carotenoid spheroidene to the red spheroidenone by incorporating a C2 keto group; here, the RC–LH1–PufX dimer:monomer ratio is altered (see below).

**Fig. 4:**
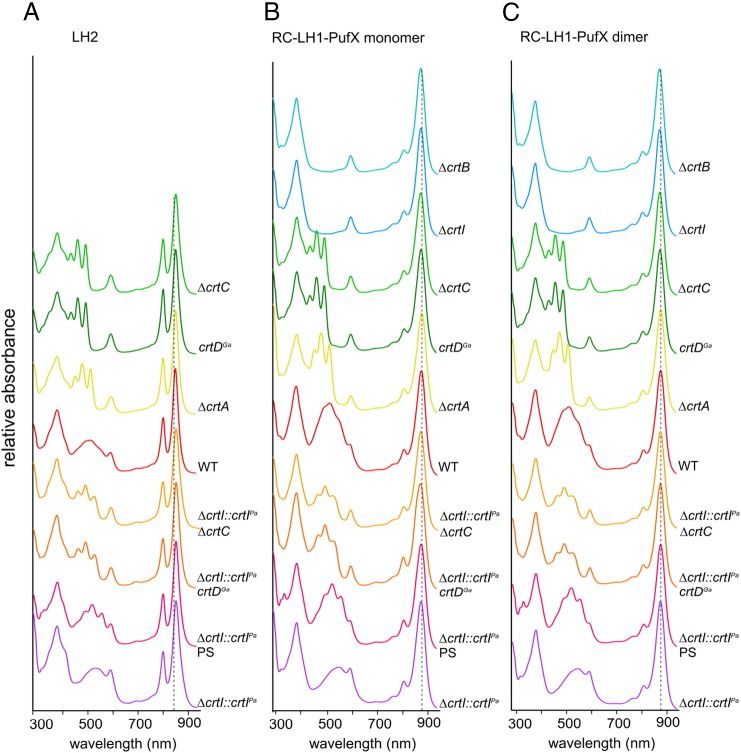
LH2 and core complex absorption spectra. (A) Stacked LH2 absorbance spectra for each carotenoid mutation; the carotenoids with the most extended conjugation length (such as 2,2′-diketo-spirilloxanthin) are nearer the bottom. Δ*crtI::crtI^Pa^* indicates a strain with the native three-step phytoene desaturase replaced by the *crtI* from *P. agglomerans* encoding the four-step enzyme. Δ*crtI::crtI^Pa^ crtD^Ga^* indicates a *crtI* replacement strain as previously described, but also incorporating the Ga mutation of the *crtD* gene. There are no spectra for the Δ*crtI* and Δ*crtB* strains, since they do not assemble LH2 complexes. (B) Absorption spectra for monomeric RC–LH1–PufX complexes, as in (A), including cores from the Δ*crtI* and Δ*crtB* strains. (C) Absorption spectra for dimeric RC–LH1–PufX complexes. All complexes were retrieved from sucrose density gradients as in Fig. 3. The complexes are from photosynthetically grown cells, with the exception of the WT and the *ΔcrtI::crtI^Pa^*, (top) samples, where semi-aerobic growth was necessary for synthesis of spheroidenone and 2,2′-diketo-spirilloxanthin, respectively.

As expected, no LH2 complexes were found for Δ*crtB* and Δ*crtI* mutants, because neurosporene or subsequent carotenoids are essential for the stable assembly of this antenna in the *Rba. sphaeroides* photosynthetic membranes [5]. LH2 complexes were present in the rest of the gradients, so the assembly pathway for this antenna can accommodate both native and non-native carotenoids. It was already established that this was the case for lycopene [11], but the efficient incorporation of rhodopin, spirilloxanthin or 2,2′-diketo-spirilloxanthin into *Rba. sphaeroides* LH2 complex underlines the permissive nature of the assembly process. To find out more about the efficiency of biosynthetic incorporation of these new carotenoids we estimated the occupancy of the pigment binding sites in LH2 complexes for each of the LH2 complexes from the samples from semiaerobically grown strains. The carotenoid:BChl ratio was measured following solvent extraction, as detailed in the Materials and methods section. Assuming a constant BChl content for these complexes, which is reasonable given the similar B800:B850 absorbance ratios in Fig. 4A, the amount of carotenoid extracted measures the extent to which these pigments are accommodated within the LH2 complexes. Table 1 shows that the carotenoid:BChl ratio is fairly constant at around 0.4–0.5, consistent with a much earlier determination [35], and that each of the non-native carotenoids is incorporated with high efficiency into the LH2 complex. The only exceptions are lycopene (0.36) and rhodopin (0.35), where slightly lower levels of these non-native carotenoids are found.

**Table 1:** Pigment content of LH2 complexes for the strains used in this study. All cultures were grown semi-aerobically in the dark with the exception of *ΔcrtI::crtI^Pa^*PS, where the cells were grown photosynthetically under anaerobic conditions to prevent conversion of spirilloxanthin to keto-spirilloxanthin. Cells of each strain were extracted and analysed as described in the Materials and methods section. Minor carotenoids 5% or below of the total are not listed. Quantification of BChls and carotenoids in LH2 complexes was performed as described in the Materials and methods section.

Strain	Carotenoid content	LH2 carotenoid:BChl
Δ*crtB*	None	N/A
Δ*crtI*	100% phytoene	N/A
Δ*crtC*	100% neurosporene	0.44 ± 0.02
*crtD^Ga^*	54% methoxy-neurosporene	0.44 ± 0.01
	34% neurosporene,	
	12% hydroxy-neurosporene	
Δ*crtA*	96% spheroidene	0.50 ± 0.04
*WT*	94% spheroidenone	0.42 ± 0.03
Δ*crtI::crtI^Pa^* Δ*crtC*	91% lycopene	0.36 ± 0.01
	9% neurosporene	
Δ*crtI::crtI^Pa^ crtD^Ga^*	34% lycopene	0.35 ± 0.01
	26% rhodopin	
	18% dimethoxy-lycopene	
	11% methoxy-lycopene	
	7% methoxy-hydroxy-lycopene	
Δ*crtI::crtI^Pa^*PS	71% spirilloxanthin	0.45 ± 0.06
	13% anhydro-rhodovibrin	
	9% lycopene	
Δ*crtI::crtI^Pa^*	62% 2,2′-diketo-spirilloxanthin	0.46 ± 0.01
	16% keto-spirilloxanthin	
	14% keto-anhydro-rhodovibrin	

The levels of monomeric and dimeric RC–LH1–PufX complexes were established by recovering the complexes from the gradients and recording absorption spectra. Slightly surprisingly, approximately 42% of the complexes were in the dimeric state in the complete absence of carotenoid (photosynthetically grown Δ*crtB* mutant; Fig. 3, #1, lower row), or with phytoene present (Δ*crtI* mutant; #2, lower row); a much lower proportion of dimers was observed in a previous analysis of the Δ*crtB* mutant R-26 [22]. In the rest of the samples RC–LH1–PufX complexes are in a largely dimeric state, with the exception of rhodopin (photosynthetic growth) and spheroidenone (semiaerobic). This latter result is significant for wild-type *Rba. sphaeroides*; comparison with either the photosynthetic, anaerobic wild-type (#5, upper row) shows how the normal dominance of the RC–LH1–PufX dimer is reversed under even limiting oxygen conditions. This is underlined by the Δ*crtA* samples (#4 upper row and #5, lower row), which abolish *crtA*-encoded spheroidene monooxygenase activity and raise the proportion of dimers as high as 82%. The new carotenoid rhodopin also favours RC–LH1–PufX dimers over monomers, but this preference is even more pronounced in the case of spirilloxanthin (#6, upper row) and 2,2′-diketo-spirilloxanthin (#7, lower row). The histograms in Fig. 3 show that the proportion of core dimers was elevated for the spirilloxanthin strain versus the spheroidene wild-type (both photosynthetically grown), and for 2,2′-diketo-spirilloxanthin versus spheroidenone (both semi-aerobically grown).

The absorption spectra of the complexes from these sucrose density gradients are presented in Fig. 4. The characteristic absorption of LH2, RC–LH1–PufX monomer and RC–LH1–PufX dimer complexes in the 800–900 nm region is retained in all cases; absorption of the B800 LH2 pigment is unaffected by the presence of lycopene, rhodopin, spirilloxanthin or 2,2′-diketo-spirilloxanthin, despite the close association of carotenoids and the B800 binding site [7]. Similarly, these new carotenoids produce no shifts in the B850 absorption of LH2 complexes, nor in the B875 nm absorption of RC–LH1–PufX monomer and dimer complexes. These results emphasise the remarkable tolerance of the LH2 and RC–LH1–PufX assembly pathways for non-native carotenoids, especially in the case of dimeric RC–LH1–PufX complexes that have a high carotenoid content of one carotenoid per LH1 BChl, i.e. 58 per complex, including the two RCs [36].

### 3.3 Analysis of carotenoids by HPLC and mass spectrometry

**Fig. 5:**
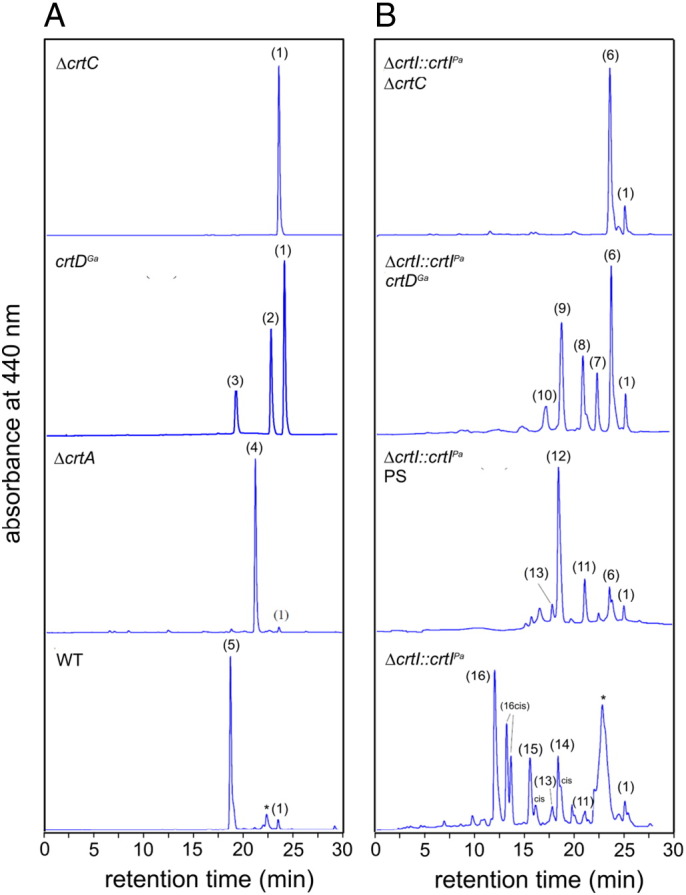
HPLC analysis of the carotenoids in LH2 complexes prepared from the various carotenoid mutants. Carotenoids were extracted and analysed as described in the Materials and methods section. The assignments are based on the MS analyses shown in Figs. 6 and S1, and Table 2 is as follows for (A) the native pathway: (1) neurosporene, (2) methoxy-neurosporene, (3) hydroxy-neurosporene, (4) spheroidene, (5) spheroidenone. (B) shows the carotenoids produced from the non-native pathway: (6) lycopene, (7) methoxy-lycopene, (8) dimethoxy-lycopene, (9) rhodopin, (10) didehydro-rhodovibrin, (11) anhydro-rhodovibrin, (12) spirilloxanthin, (13) didehydro-rhodopin, (14) keto-anhydro-rhodovibrin, (15) keto-spirilloxanthin, (16) 2,2′-diketo-spirilloxanthin. The asterisks indicate BChl *a* and BPhe *a*.

**Fig. 6:**
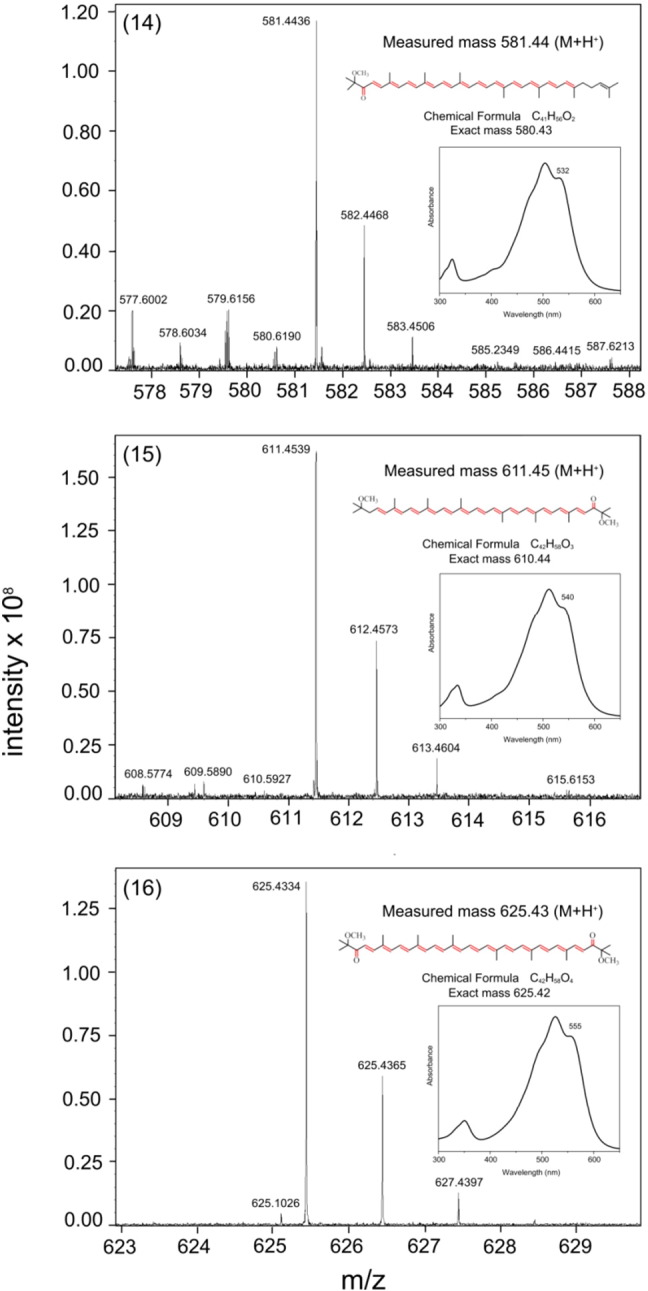
Mass spectrometry of carotenoids corresponding to peaks 14–16 in the HPLC analysis. Each panel shows the MS analysis, the measured mass, structure, chemical formula and exact mass of the carotenoid. Each inset absorption spectrum is taken from the HPLC analysis. (14) is keto-anhydro-rhodovibrin, (15) is keto-spirilloxanthin and (16) is 2,2′-diketo-spirilloxanthin.

The carotenoids were extracted from LH2 complexes prepared from membranes purified from semiaerobically grown cells, then analysed by HPLC (Fig. 5). The carotenoid fractions were subsequently analysed by mass spectrometry (MS); all MS data, together with HPLC analyses, are displayed in Fig. S1, and summarised in Table 2. The only omission is methoxy-neurosporene, peak 2 in the HPLC analysis, where the MS spectrum was of poor quality. The presence of this carotenoid in *crtD* mutants has been well established [10]. Three examples of the MS analysis are shown in Fig. 6, for keto-anhydrorhodovibrin, keto-spirilloxanthin and 2,2′-diketo-spirilloxanthin. Overall these analyses demonstrate the conversion of lycopene, produced by the *crtI^Pa^*-encoded four-step phytoene desaturase, to rhodopin and eventually to spirilloxanthin (anaerobic photosynthetic growth) or 2,2′-diketo-spirilloxanthin (semiaerobic growth). Thus, the native CrtC, D, F and A enzymes are unable to detect the earlier degree of phytoene desaturation and they modify lycopene and other carotenoids efficiently, as if they were native pigments. The presence of minor carotenoids in *ΔcrtI::crtI^Pa^ crtD^Ga^* and *ΔcrtI::crtI^Pa^* strains is discussed in Section 4.1.

**Table 2:** Summary of assignments from mass spectrometry analysis of carotenoids 1–15 in Fig. 5. A full summary is shown in Fig. S1.

Carotenoid	HPLC peak number	Chemical formula	Exact mass	Measured mass (M + H^+^)	Structure
Neurosporene	1	C_40_H_58_	538.45	539.47	
Hydroxy-neurosporene	3	C_40_H_60_O	556.46	557.47	
Spheroidene	4	C_41_H_60_O	568.46	569.47	
Spheroidenone	5	C_41_H_58_O_2_	582.44	583.45	
Lycopene	6	C_40_H_56_	536.44	537.44	
Methoxy-lycopene	7	C_41_H_60_O	568.46	569.47	
Dimethoxy-lycopene	8	C_42_H_64_O_2_	600.49	601.49	
Rhodopin	9	C_40_H_58_O	554.45	555.45	
Methoxy-hydroxy-lycopene	10	C_41_H_62_O_2_	586.47	587.48	
Anhydro-rhodovibrin	11	C_41_H_58_O	566.45	567.45	
Spirilloxanthin	12	C_42_H_60_O_2_	596.46	597.48	
Didehydro-rhodopin	13	C_41_H_56_O	552.43	553.45	
Keto-anhydro-rhodovibrin	14	C_41_H_56_O_2_	580.43	581.44	
Keto-spirilloxanthin	15	C_42_H_58_O_3_	610.44	611.45	
2,2′-Diketo-spirilloxanthin	16	C_42_H_58_O_4_	625.42	624.43

### 3.4 Estimations of energy transfer efficiency from fluorescence spectroscopy

**Fig. 7:**
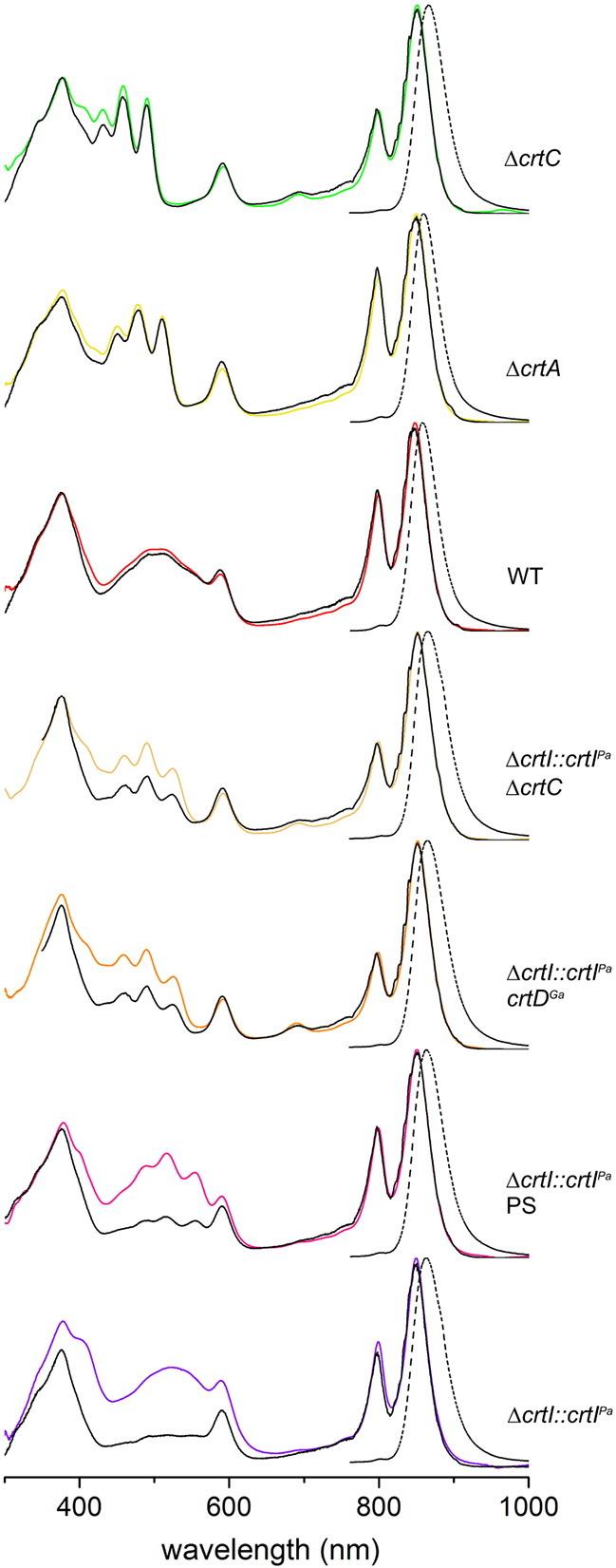
Analysis of carotenoid–BChl energy transfer in purified LH2 complexes using fluorescence and absorption spectroscopy. The fluorescence spectra (dashed), corrected for the instrument response, were obtained using excitation of the BChls at 590 nm; spectra with the same shapes were obtained at other excitation wavelengths, including exciting the carotenoid (450–570 nm). A weak 1/λ^4^ light-scattering correction has been applied to the absorptance (1-transmittance) spectra (coloured), which primarily affects the spectra at λ < 550 nm. Fluorescence excitation spectra (black) were obtained by monitoring the emission from the B850 BChls at 900 (or 910) nm and scanning the excitation wavelengths between 350 and 1000 nm. In some cases, a small feature at 900 (or 910) nm due to scattered excitation light in the emission channel was observed and was digitally removed from the fluorescence excitation spectrum for clarity. The fluorescence excitation spectra were monitored at several wavelengths from 900 to 910 and then averaged. Pairs of fluorescence excitation (black) and absorptance (coloured) spectra were normalised at 850 nm (Q_y_ band). The efficiency of energy transfer was obtained by measuring the excitation/absorptance amplitude (peak-height) ratio at two wavelengths in the 470–550 nm range and averaging.

Absorptance, fluorescence excitation and fluorescence emission spectra were recorded in order to compare the ability of native and non-native carotenoids to transfer absorbed energy to the BChl pigments within the LH2 complexes. Fig. 7 shows the paired absorptance and excitation spectra for a series of the LH2 complexes with the native carotenoids neurosporene, spheroidene, spheroidenone and the non-native lycopene, rhodopin, spirilloxanthin and 2,2′-diketo-spirilloxanthin. The absorptance and excitation spectra were normalised at the BChl 850 nm Q_Y_ maximum. In most cases it was also possible to obtain acceptably normalised spectra in the BChl 590 nm Q_x_ and BChl 375 nm Soret bands. The gradually red-shifted carotenoid absorption, a consequence of the increasing conjugation length, *N*, encroaches significantly on the BChl Q_x_ band, especially for spheroidenone, spirilloxanthin and 2,2′-diketo-spirilloxanthin. In some such cases, normalising absorptance and fluorescence excitation spectra at 850 nm did not yield spectra also normalised at 590 nm, because of spectral overlap from carotenoids in the 500–600 nm region. For these reasons, normalisation of the absorptance and excitation spectra at 850 nm, rather than the 590 nm more commonly used, was employed in all cases as the measure of 100% energy transfer efficiency. The efficiency of energy transfer from carotenoid to BChl (leading to emission monitored from the 850 nm BChls), was obtained by taking the excitation/absorptance amplitude (peak-height) ratio at two wavelengths in the carotenoid contour (470–550 nm) where there is minimal overlap with the BChl Soret or Q_x_ features, and then averaging. Analysis of the data in Fig. 7 reveals a clear trend, with a high energy transfer efficiency of 94% for neurosporene (*N* = 9), 96% for spheroidene (*N* = 10), 95% for spheroidenone (*N* = 11), 64% for lycopene (*N* = 11), 62% for rhodopin (*N* = 11), 39% for spirilloxanthin (*N* = 13) and 35% for 2,2′-diketo-spirilloxanthin (*N* = 15). The high value for the N = 11 carotenoid spheroidenone does not fit this pattern; possible reasons for this apparent anomaly will be discussed.

## 4 Discussion

### 4.1 Synthesis of the spirilloxanthin series of carotenoids in *Rba. sphaeroides*

In a previous study [11], we reported the construction and properties of *Rba. sphaeroides* strains that incorporated lycopene into LH2 and RC–LH1–PufX complexes, achieved initially by inactivating the native 3-step phytoene synthase gene using directed Tn*5* transposition [37], [27]. Subsequently, the *Erwinia herbicola* (now *P. agglomerans*) *crtI* gene, designated *crtI^Pa^* in the present study, was transferred to this *crtI*::Tn*5* mutant using the broad host range vector pRKSK1 [38]. This work also made use of an undefined point mutation in *crtC*, which, when coupled with the plasmid-borne expression of the four-step *P. agglomerans crtI*, halted the new spirilloxanthin pathway at an early stage. Lycopene, the dominant (91%) carotenoid in this new strain, entered the assembly pathway for photosynthetic complexes, and transferred absorbed light energy to BChls [11].

This early example of synthetic biology relied on slightly cumbersome genetic modifications, with genomic insertion of Tn*5* into *crtI* exerting polar effects on the adjacent *crtB* gene [33], and plasmid-borne expression of the *P. agglomerans crtI* gene driven by the strong *puc* promoter [38] likely driving abnormal levels of transcription and enzyme production. Thus, the normal balance of enzyme levels would have been disrupted, with possible effects on flux down the carotenoid pathway. The present work uses pK18mob*sacB* [16] to seamlessly integrate foreign genes into the *Rba. sphaeroides* chromosome. Thus, serial genetic alterations avoid the increasing metabolic cost of antibiotic resistance genes, multiple polar effects on nearby genes, and use native promoters to maintain the level of transcription of pathway genes. The stability of these strains gives them the outward appearance of new bacteria, with hybrid properties conferred by new carotenoids assembling into native antenna and reaction centre complexes.

**Fig. 8:**
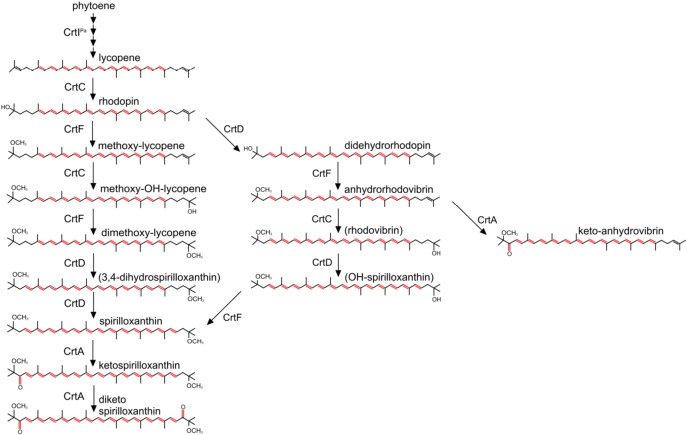
Possible non-native biosynthetic pathways to 2,2′-diketo-spirilloxanthin that account for most of the carotenoids identified in Fig. S1. The carotenoids labelled in parentheses have not been identified. Carbon–carbon double bonds are labelled in red.

Using the three/four-step phytoene desaturase swap as a baseline, further deletions were used to halt the spirilloxanthin pathway at the level of lycopene or rhodopin, with the absence of oxygen in anaerobic photosynthetic cultures restricting conversion of spirilloxanthin to 2,2′-diketo-spirilloxanthin. The presence of these carotenoids in various strains confirms earlier studies, which proposed that the type of phytoene desaturase is a major control point, determining whether the spheroidene or spirilloxanthin series of carotenoids is synthesised [15]. However, other interactions also determine the type of carotenoid; for example the native CrtC enzyme can intervene in the sequence of four desaturations, prematurely terminating the reaction sequence catalysed by the *P. agglomerans* CrtI, and approximately 20% of the flux is then directed down the spheroidene pathway [11]. The absence of CrtC in the present work ensures the sequence of desaturations goes to completion and all the carotenoid enters the spirilloxanthin route. Thus the Δ*crtI::crtI^Pa^* Δ*crtC*, Δ*crtI::crtI^Pa^*PS and *crtI::crtI^Pa^* (semi-aerobic) strains synthesise mainly lycopene, spirilloxanthin and 2,2′-diketo-spirilloxanthin, respectively. In this last case, careful manipulation of the growth conditions, for example increasing aeration, could increase the levels of 2,2′-diketo-spirilloxanthin still further. Ideally, the new Δ*crtI::crtI^Pa^ crtD^Ga^* strain would have one dominating carotenoid, namely rhodopin, but the HPLC and MS analyses (Fig. 5, Fig. 6; Table 1, Table 2; Fig. S1) show that this strain contains appreciable quantities of other minor carotenoids such as rhodopin, dimethoxy-lycopene, methoxy-lycopene and methoxy-hydroxy-lycopene. Although they are undesirable from a synthetic biology perspective the identification of minor carotenoids did allow the compilation of a plausible pathway for the biosynthesis of 2,2′-diketospirilloxanthin in *Rba. sphaeroides* (Fig. 8), which takes into account the carotenoid biosynthetic intermediates recorded in the HPLC and MS analyses in Fig. 5, Fig. 6, and S1, and summarised in Table 1, Table 2. We consider that the pathway including dimethoxy-lycopene, methoxy-lycopene and methoxy-hydroxy-lycopene dominates, since only one of the carotenoids, 3,4-dihydro-spirilloxanthin, was not detected, possibly because it is a transient intermediate during the sequential two-step conversion of dimethoxy-lycopene to spirilloxanthin catalysed by CrtD (Fig. 8).

This new pathway differs from the normal spirilloxanthin pathway (lycopene, rhodopin, 3,4,-didehydro-rhodopin, anhydro-rhodovibrin, rhodovibrin, hydroxy-spirilloxanthin, spirilloxanthin) found in *Rsp. rubrum* [10]. This difference could arise because the native CrtC, D, F and A enzymes have to deal with unfamiliar substrates, and possibly CrtF catalyses the methylation of rhodopin more readily than CrtD desaturates it (Fig. 8).

The Δ*crtI::crtI^Pa^* strain produces approximately 16% keto-spirilloxanthin, with the assumption that this keto group is at the same C2 position as in the native keto carotenoid, spheroidenone (see Figs. 6, S1). The mode of action of spheroidene monooxygenase (CrtA), which also acts as a spirilloxanthin monooxygenase, is unknown but apparently CrtA has access to both ends of its carotenoid substrate, allowing the formation of 2,2′-diketo-spirilloxanthin. Curiously, we see no evidence in the semiaerobically grown wild-type for the presence of 2,2′-diketo-spheroidene, that is spheroidenone with an extra keto group at the 2′ position. The way in which CrtA is presented with native and non-native substrates is worthy of further investigation.

### 4.2 Functional assembly of lycopene, rhodopin, spirilloxanthin and 2,2′-diketo-spirilloxanthin into LH2 and RC–LH1–PufX complexes

The earlier work of Garcia-Asua et al. [11] showed that lycopene is efficiently incorporated into LH and RC complexes. LH2 assembly requires visibly coloured carotenoids, from neurosporene onwards [5], so the ability of lycopene to substitute for spheroidene is perhaps not surprising. The present work further tests the adaptability of the photosystem assembly pathways in *Rba. sphaeroides*, and we find no obstacles to incorporation of lycopene, rhodopin, spirilloxanthin or 2,2′-diketo-spirilloxanthin into either LH2 or RC–LH1–PufX complexes. Indeed, the presence of minor carotenoids such as methoxy-lycopene, dimethoxy-lycopene, methoxy-hydroxy-lycopene, anhydro-rhodovibrin, didehydro-rhodopin and keto-anhydrorhodovibrin in purified LH2 complexes underlines the lack of specificity of carotenoid binding sites in this antenna, and for RC–LH–PufX as well.

Inspection of the sucrose density gradients in Fig. 3, all loaded for equal LH1 content, shows little variation in the LH2 levels, with the obvious exception of the Δ*crtB* and Δ*crtI* mutations, which are known to prevent LH2 assembly. Similarly, the balance between monomeric and dimeric RC–LH1–PufX complexes is only affected in the lycopene membranes from photosynthetically grown cells, and the spheroidenone membranes from wild-type semiaerobically grown cells. The occupancies of the LH2 binding sites for the non-native carotenoids lycopene, rhodopin, spirilloxanthin and 2,2′-diketo-spirilloxanthin are comparable with those for the native neurosporene, spheroidene and spheroidenone (Table 1) reinforcing the notion that the LH2 assembly pathway tolerates a wide variety of these pigments. This pathway also shows some tolerance for BChls esterified with geranylgeraniol, rather than with phytol as in the WT; a Tn5 insertion into *bchP*, encoding the geranylgeraniol reductase did significantly reduce levels of photosystem complexes but despite the 70% reduction in the B800 absorbance of whole cells, largely attributable to LH2, approximately 65% of the B850 absorbance was retained, red-shifted by approximately 6 nm relative to the WT [39].

### 4.3 The effects of carotenoid conjugation length on carotenoid-to-bacteriochlorophyll energy transfer in LH2 complexes assembled in vivo

There have been several studies of the relationship between carotenoid conjugation length on carotenoid–BChl energy transfer, many relying on *in vitro* reconstitutions. This elegant approach enables the use of non-natural carotenoids, synthesised chemically and customised to extend the conjugation length stepwise from 7 to 13 carbon–carbon double bonds, one at a time. Frank and co-workers [17] examined a series of spheroidene analogues: 3,4,7,8-tetrahydrospheroidene (*N* = 7), 3,4,5,6-tetrahydrospheroidene (8), 3,4-dihydrospheroidene (9), spheroidene (10), 5′,6′-dihydro-7′,8′-didehydrospheroidene (11), 7′,8′-didehydrospheroidene (12), and 1′,2′-dihydro-3′,4′,7′,8′-tetradehydrospheroidene (13). Their data show a correlation between *N* and carotenoid-to-BChl energy transfer efficiency, which drops from 95 to 12% over the series of carotenoids studied. The extent of incorporation of these carotenoids into an initially carotenoid–less LH2 complex varied between 100 and 52%, so the use of *in vivo*-assembled complexes can in principle offer a more efficiently packaged set of carotenoids. The studies mentioned above, as well as others, for example [40], [13], [41] used a carotenoid–less LH2 as the recipient of these and other carotenoids, which were added to the complex in solvent. This complex was prepared from R26.1, a partial revertant of the original R26, now known to be a *crtB* mutant [22] that harbours a Phe → Val mutation in the LH2α polypeptide, as well as lacking the N-formyl Met ligand to the B800 BChl [42]. The use of WT LH2 polypeptides to bind a range of carotenoids avoids the use of the R26.1 background, and further variations of the system are now possible, including combinations of different carotenoids with mutations that are already known to perturb carotenoid binding sites. For example, the βArg-10 → Glu mutation blue-shifts carotenoid absorbance maxima by approximately 6 nm and greatly attenuates the electrochromic response of LH2 carotenoids to an external membrane potential [43]. To underline the close association between LH2 carotenoids and the B800 binding site, these changes are accompanied by blue-shifted and broadened the B800 BChl absorption [44], [45]; a subsequent FT-Raman study showed that this Arg residue forms an H-bond with the C2 acetyl carbonyl of the monomeric BChl in LH2 [46]. Another mutation, βHis-18 → Ser, nearly abolishes B800 absorption and also affects carotenoid absorption and electrochromic behaviour [43]. Interestingly, each of these mutants has a normal carotenoid content, and energy transfer to B850 pigments is unaffected [43]. This work was performed in a neurosporene background, but can now be revisited with other carotenoids present. The present study shows that absorption of the B800 LH2 pigment is unaffected by the presence of the non-native lycopene, rhodopin, spirilloxanthin or 2,2′-diketo-spirilloxanthin, despite the close association of carotenoids and the B800 binding site [44], [45].

**Fig. 9:**
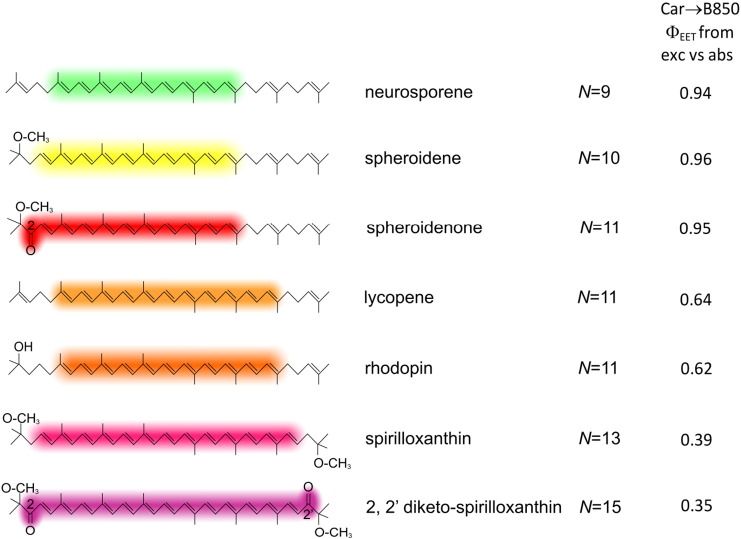
Diagram showing the conjugation length, *N*, represented in the structures of the various carotenoids by diffuse coloured bands, and the carotenoid–BChl energy transfer efficiencies for the various LH2 complexes studied.

The data in Fig. 9 reveal a clear trend, with a high energy transfer efficiency of 94% for neurosporene (*N* = 9), 96% for spheroidene (*N* = 10), 95% for spheroidenone (*N* = 11), 64% for lycopene (*N* = 11), 62% for rhodopin (*N* = 11), 39% for spirilloxanthin (*N* = 13) and 35% for 2,2′-diketo-spirilloxanthin (*N* = 15). The expected correlation between *N* and carotenoid–BChl energy transfer efficiency extends previous studies by two conjugated double bonds. Energy transfer has dropped to 35% for *N* = 15, so it would be interesting to measure the intrinsic lifetimes of the S_2_ and S_1_ excited states for this carotenoid, both isolated in solvent and assembled within the LH2 complex. The inclusion of the rhodopin–LH2 and other complexes in such a study would be helpful for studying the link between *N* and energy transfer dynamics, which previously relied on comparisons between *Rba. sphaeroides* and *Rps. acidophila* LH2 complexes, which have different carotenoids [14], [23]. The series of LH2 complexes in the present study also circumvents the problems of comparing the behaviour of spirilloxanthin (*N* = 13) and other carotenoids, since spirilloxanthin is generally measured using the LH1 complex of *Rsp. rubrum*. The 39% efficiency for this carotenoid in its LH2-bound state is higher than the 32% measured for the native LH1-bound spirilloxanthin [13] and 28% [19] or 36% [18] for the reconstituted complex. The figure of 65% for lycopene is marginally higher than the 54% found for the *Rba. sphaeroides* lycopene–LH2 complex engineered in an earlier study [11], although 27% was found the *Rsp. molischianum* LH2 complex [23]. Finally, 62% energy transfer efficiency for rhodopin is also higher to the 53% found for rhodopin glucoside in the LH2 complex from *Rps. acidophila* 10050 [14], and much higher than the 29% for rhodopin reconstituted into the LH2 complex of *Chromatium vinosum* [13]. The values of 94–96% for neurosporene, spheroidene, and spheroidenone are in excellent agreement with the values of 91–98% obtained previously in *Rba. sphaeroides* strains [14], which used the same absorptance versus fluorescence excitation spectral analysis employed here as well as measuring the dynamics of the carotenoid S_1_ and S_2_ excited states. Each type of energy-transfer measurement has advantages and disadvantages, with spectral overlap between BChl and the longer chain carotenoids such as spirilloxanthin and 2,2′-diketo-spirilloxanthin being a hindrance for the static spectral method. Energy transfer dynamics will be explored in future measurements of the new strains containing these longer carotenoids. Measurement of BChl Q_X_ to carotenoid S_1_ backward singlet energy-transfer, followed by the energy dissipation from the carotenoid S_1_ state, would also be helpful in establishing pathways of energy transfer for mutants containing spirilloxanthin and 2,2′-diketo-spirilloxanthin, as it was for the *Rsp. rubrum* RC-LH1 complex [47].

Reconstitution studies with the LH1 complex have also demonstrated the relationship between *N* and carotenoid–BChl energy transfer. Akahane et al. [18] reconstituted neurosporene, spheroidene, lycopene, anhydrorhodovibrin and spirilloxanthin into a LH1 complex purified from the carotenoid-less G9 mutant of *Rsp. rubrum*. They used subpicosecond time-resolved absorption spectroscopy to show that with increasing *N* the efficiencies of carotenoid–BChl energy transfer were 78%, 75%, 46%, 40% and 36%, respectively. In a subsequent reconstitution study, also using the *Rsp. rubrum* LH1 complex, the efficiencies for spheroidene, rhodopin, anhydrorhodopin and spirilloxanthin were 65%, 45%, 41%, and 28% respectively [19]. It should be mentioned that reconstitution studies suffer from some drawbacks such as variable efficiency of incorporation [17] but they do allow one type of carotenoid to be studied at a time, for example for dissecting the thermodynamics of assembly of the LH1 complex [48]. The more biological approach adopted in the present study is valuable, although it also suffers from a different problem in that the pathway cannot always deliver a single carotenoid to the assembly machinery, in the case of rhodopin and 2,2′-diketo-spirilloxanthin at least. The LH2 complex assembled in the *crtD^Ga^ crtI::crtI^Pa^* strain contains 34% lycopene, 26% rhodopin, 18% dimethoxylycopene, 11% methoxylycopene and 7% methoxy-hydroxy-lycopene, but all of these carotenoids are *N* = 11 so there is no effect on the overall carotenoid–BChl energy transfer efficiency. The LH2 complexes of the Δ*crtI::crtI^Pa^* strain contain 62% 2,2′ diketo-spirilloxanthin (Table 1) but also carotenoids of slightly shorter conjugation length, namely 16% keto-spirilloxanthin and 14% keto-anhydro-rhodovibrin, so the 35% value for the carotenoid–BChl energy-transfer efficiency ascribed to 2,2′ diketo-spirilloxanthin is likely to be a slight overestimate.

As already mentioned, the high value of 95% for the *N* = 11 carotenoid spheroidenone does not conform to the trend for the other carotenoids in this study, particularly since the other N = 11 carotenoids, lycopene and rhodopin, have efficiencies in the 60–65% range. For simplicity we considered that each addition of a C=O group for spheroidenone or 2,2′-diketo-spirilloxanthin represents an incremental increase in the value of *N*, by analogy with the addition of a C=C bond to the *π*-conjugated carbon–carbon double bond system. However, the addition of C=O and C=C will not have the same effects on the electronic structure and excited-state manifolds of a carotenoid (e.g., the nature and energies of the states) or on the interactions of these pigments with the protein. Raman spectroscopy would reveal more information about the vibrational/electronic characteristics and conformation of (keto)carotenoids in LH2 and LH1 complexes. In the case of spheroidene and spheroidenone it was shown that spheroidene molecules within RC–LH1–PufX complexes form a relatively long-lived triplet state that prevents quenching of either triplet BChl or singlet oxygen, whereas the presence of spheroidenone initiates a new energy transfer pathway that promotes quenching [9]. It was suggested that upward movement of *Rba. sphaeroides* in the water column from a low-light, anaerobic environment to high-light, oxygenated surroundings would necessitate the photoprotection afforded by the oxygen-mediated conversion of spheroidene to spheroidenone [9]. Such comparisons between the functional characteristics of spheroidenone and spheroidene are also now possible between the longer-chain analogues spirilloxanthin and 2,2′-diketo-spirilloxanthin. Such studies will give further insights into the origins of the generally lower energy transfer efficiencies of the longer-chain carotenoids and differences engendered by the addition of C=O versus C=C groups to the carotenoid skeleton.

This new series of natively assembled LH2 and RC–LH1–PufX complexes with neurosporene, hydroxy-neurosporene, spheroidene, spheroidenone, lycopene, rhodopin, spirilloxanthin and 2,2′-diketo-spirilloxanthin provides a new experimental platform for investigating the effects of carotenoid variants on their conformation within LH binding sites, coupling to BChls, excited state energies and ultrafast dynamics. The apparently variable effects of *N* on carotenoid–BChl energy transfer efficiency in the literature reflect the different spectroscopic methods used to quantify this process, the use of native or reconstituted LH complexes, variations in the efficiency of reconstitution, and the type of complex studied, whether LH2 or LH1. Nevertheless the same trend is always observed with the efficiency decreasing as *N* increases [49]. The set of strains presented here allows a direct comparison of the roles of different carotenoids in a naturally assembled, consistent antenna background. Further insights into these processes will be gained by combining carotenoid variants with site-directed mutations that tune the local electrostatic environment of these newly-introduced pigments.

The following are the supplementary data related to this article.

Fig. S1 Mass spectrometry of carotenoids corresponding to peaks 1, 3–16 in the HPLC analysis shown in Fig. 5. Each panel shows the MS analysis, the measured mass, structure, chemical formula and exact mass of the carotenoid. Each inset absorption spectrum is taken from the HPLC analysis.
