# Supplementary material for: Assembly of functional photosystem complexes in Rhodobacter sphaeroides incorporating carotenoids from the spirilloxanthin pathway
Source: Biochim Biophys Acta Bioenerg. 2015 Feb;1847(2):189–201. doi: 10.1016/j.bbabio.2014.10.004 (PMC4331045; doi:10.1016/j.bbabio.2014.10.004)

Figure S1. Mass spectrometry of carotenoids corresponding to peaks 1, 3-16 in the HPLC analysis shown in Fig. 5. Each panel shows the MS analysis, the measured mass, structure, chemical formula and exact mass of the carotenoid. Each inset absorption spectrum is taken from the HPLC analysis.

Peak 1: Neurosporene

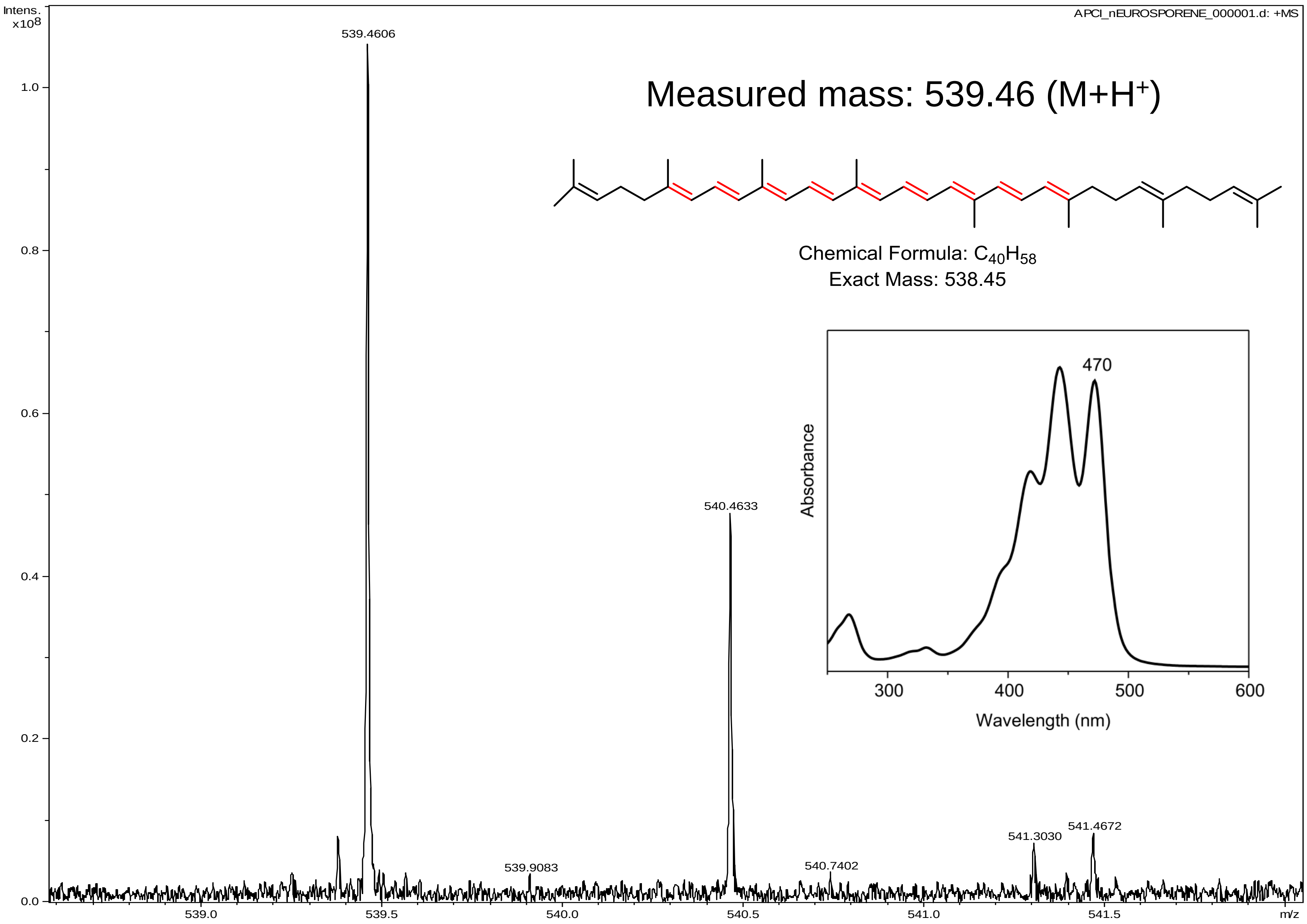

Peak 3: Hydroxy-neurosporene

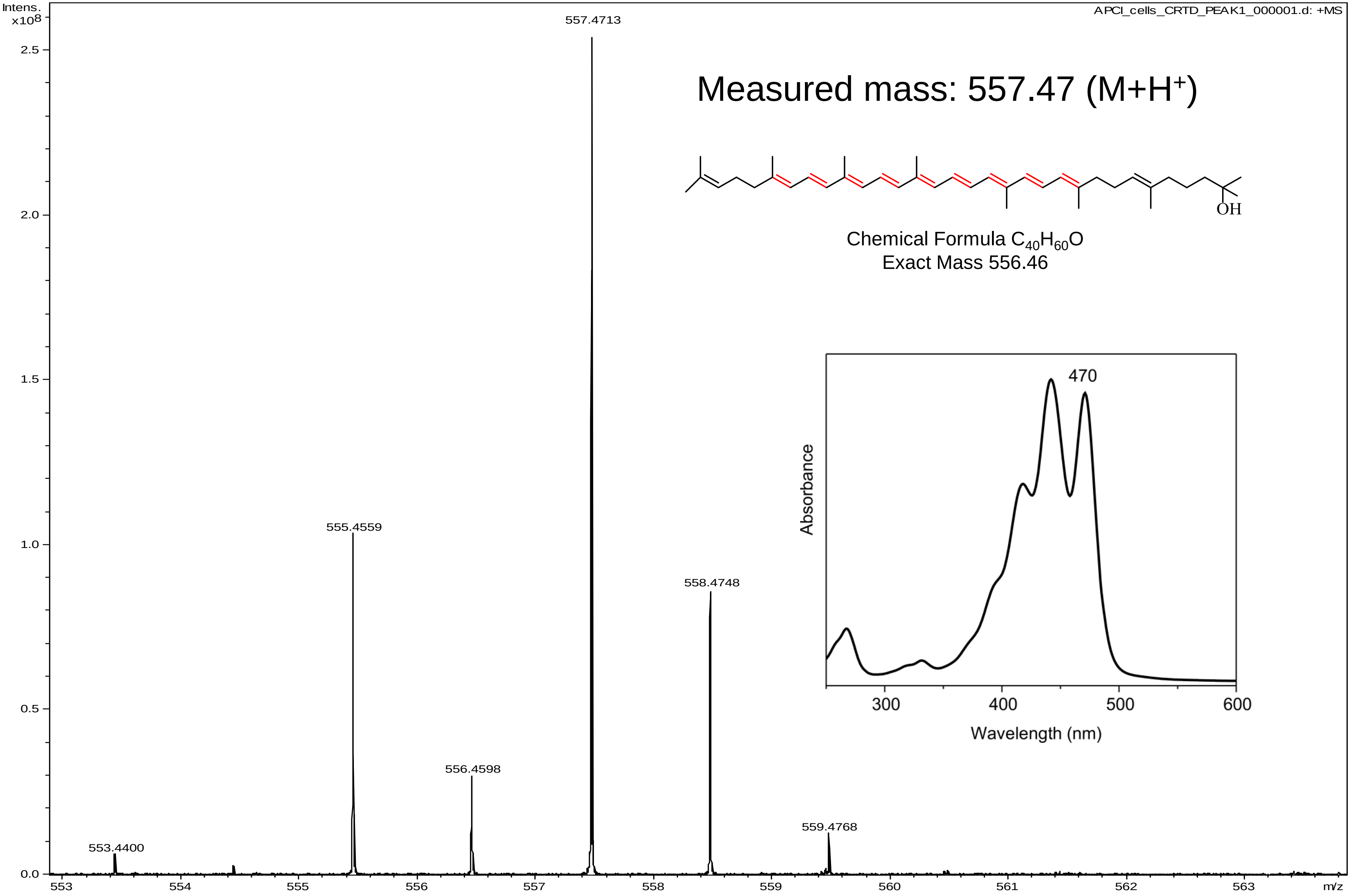

Peak 4: Spheroidene

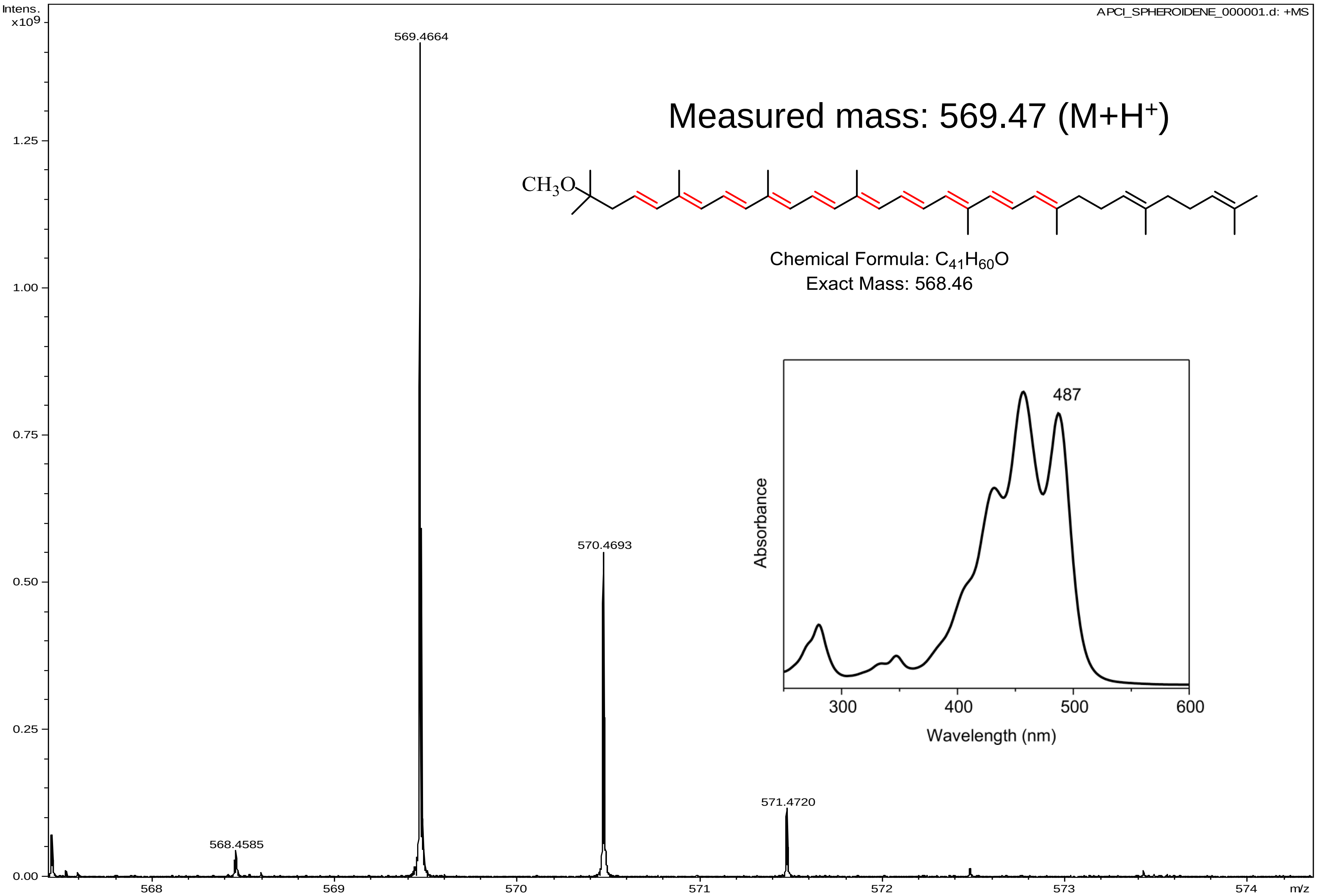

Peak 5: Spheroidenone

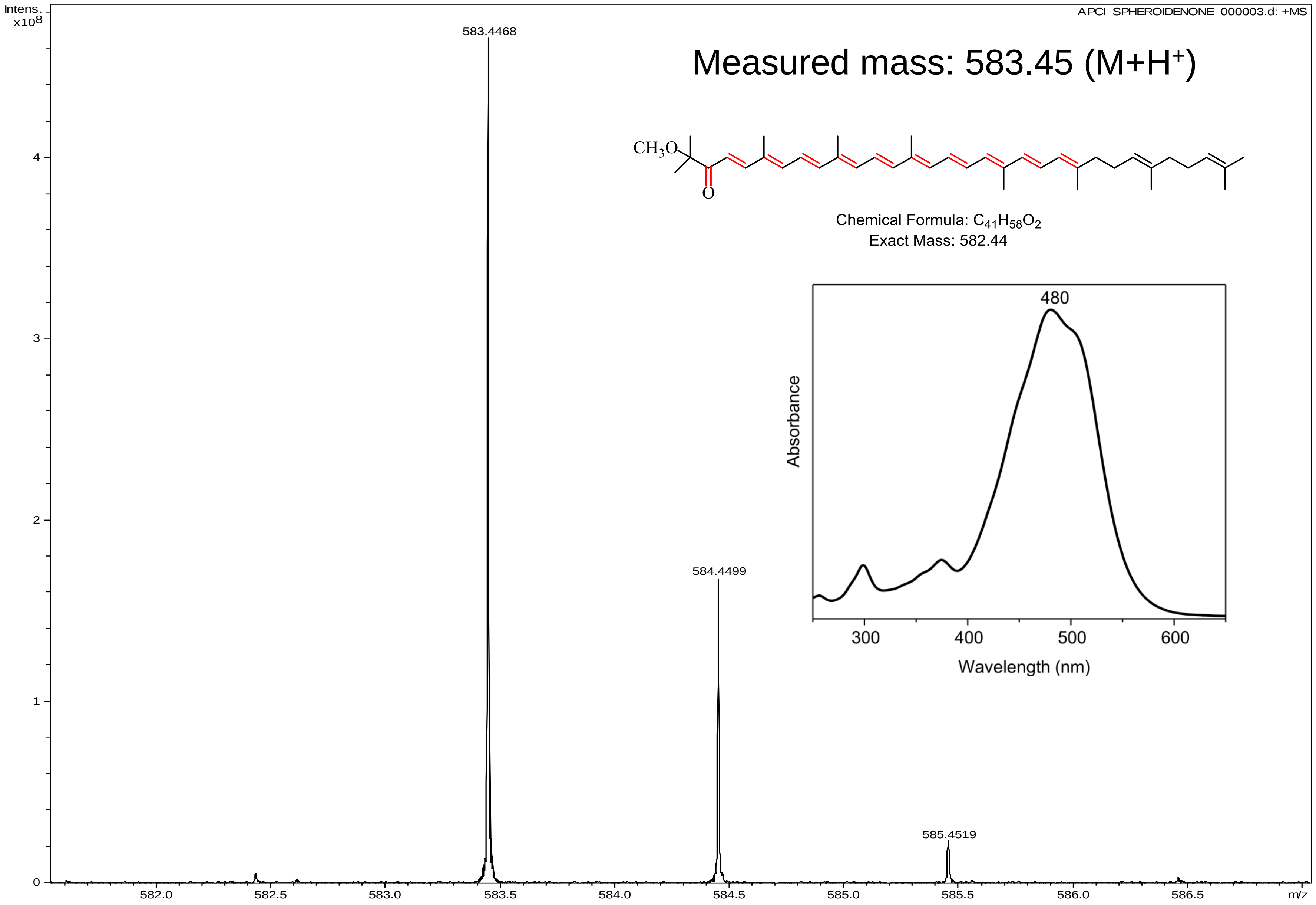

Peak 6: Lycopene

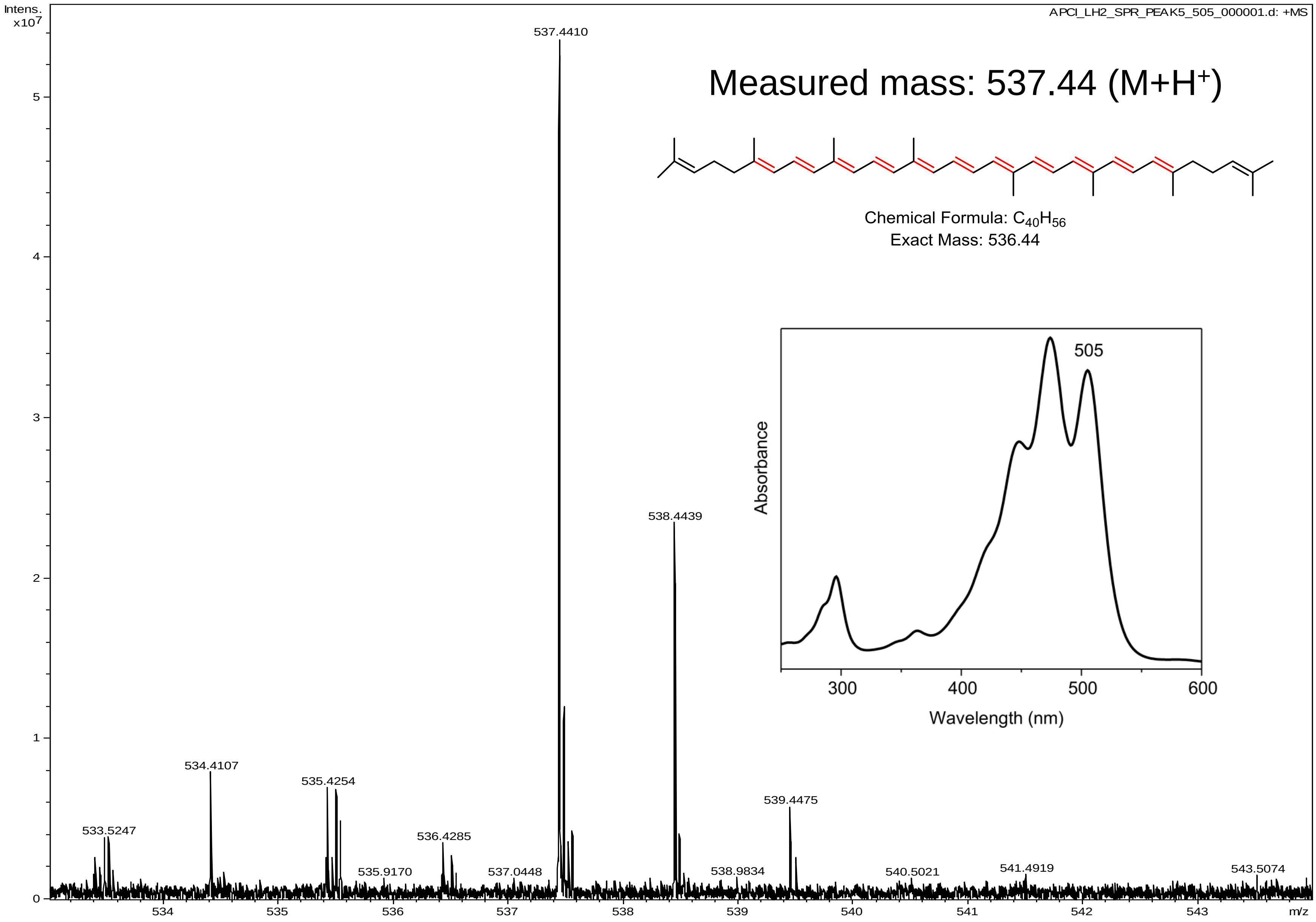

Peak 7: Methoxy-lycopene

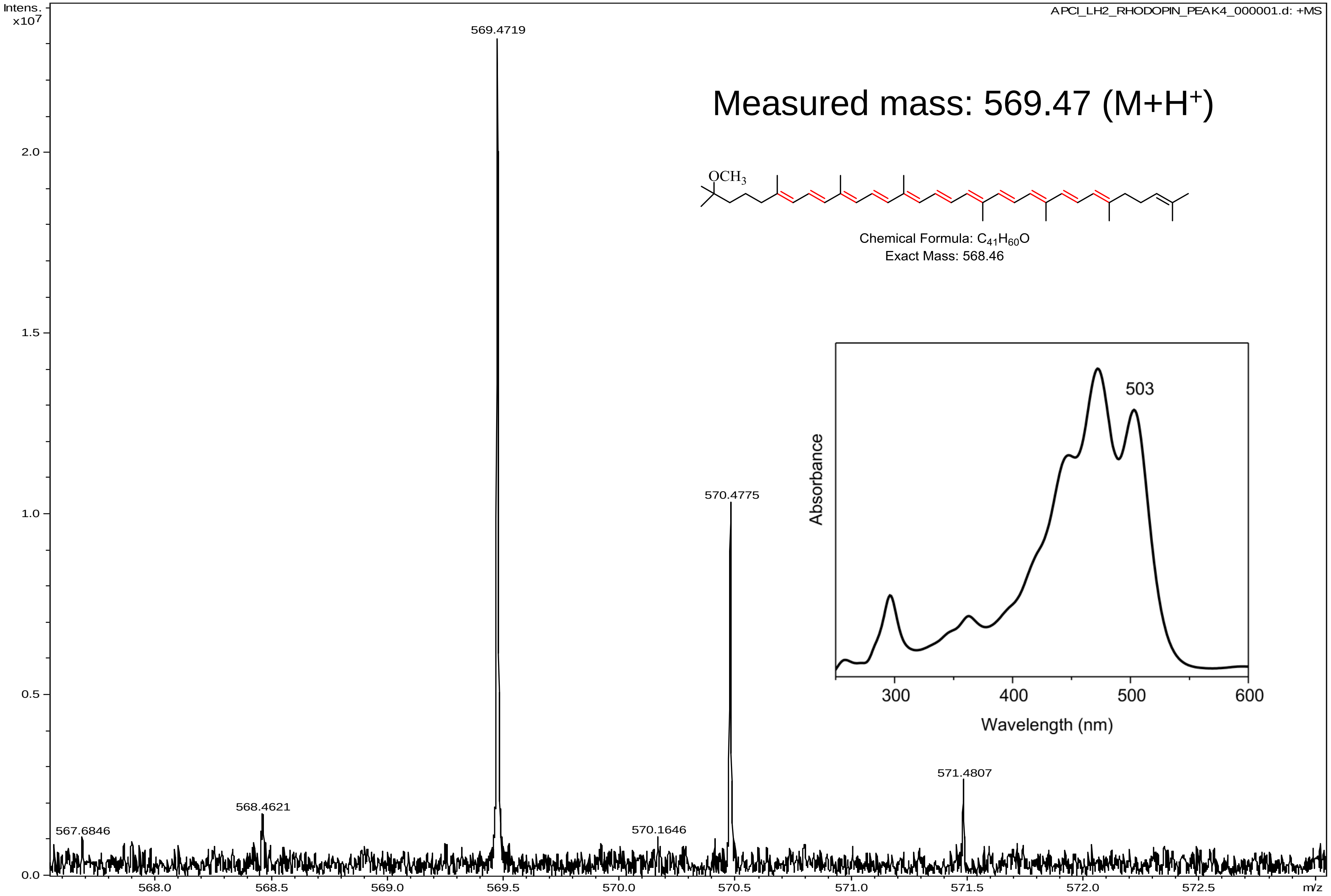

Peak 8: Di-methoxy-lycopene

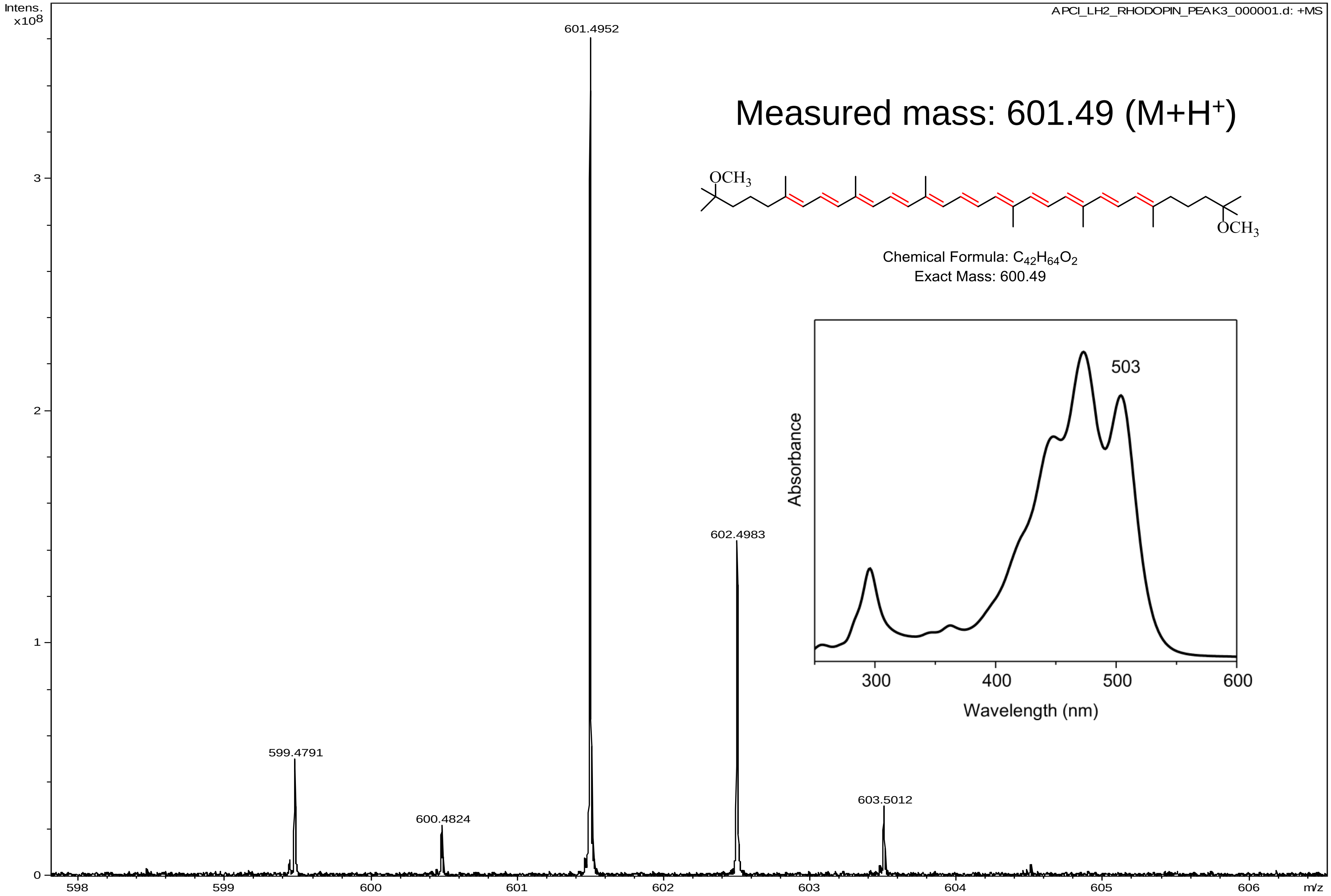

Peak 9: Rhodopin

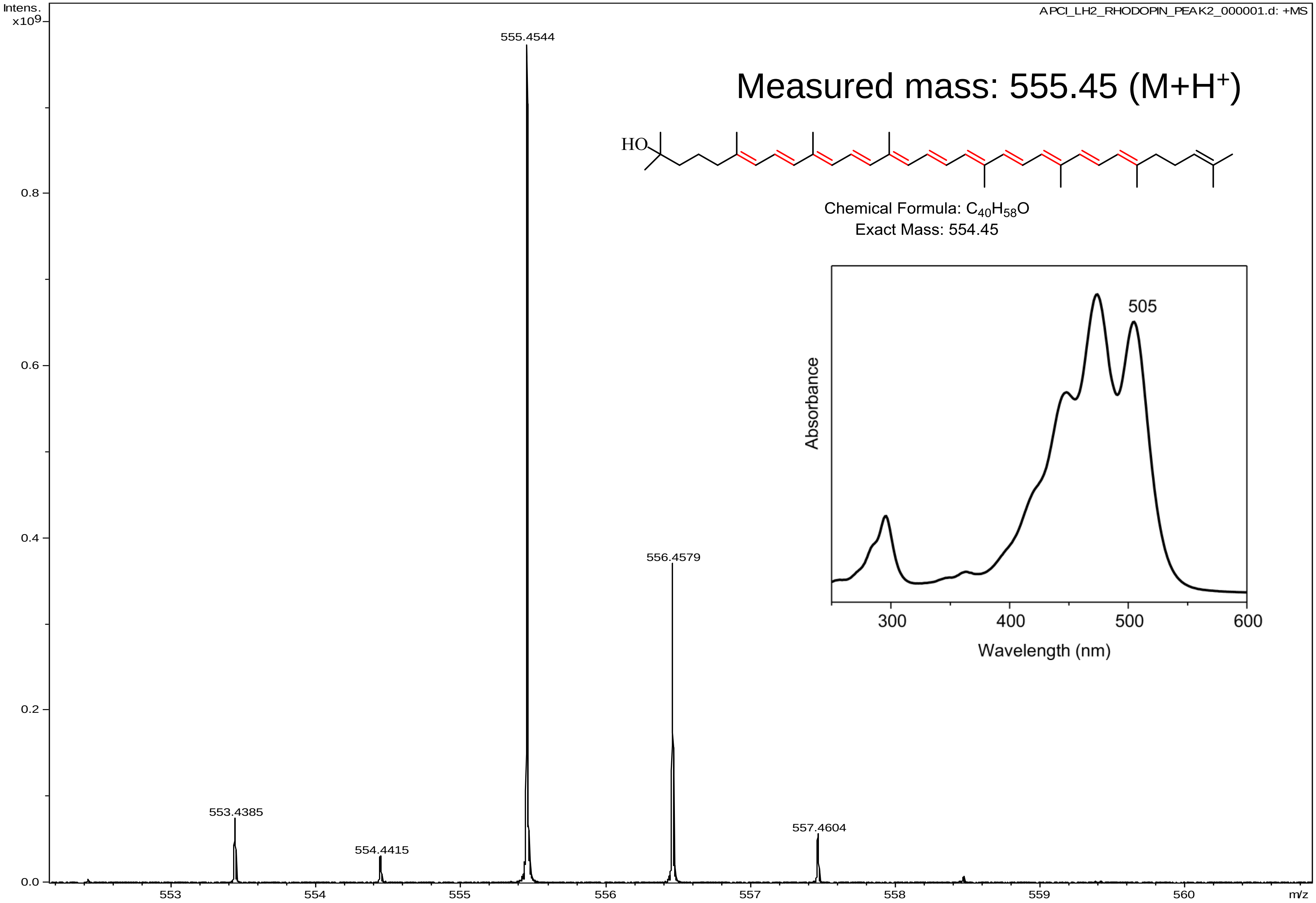

Peak 10: Di-dehydro-rhodovibrin (methoxy-hydroxy-lycopene)

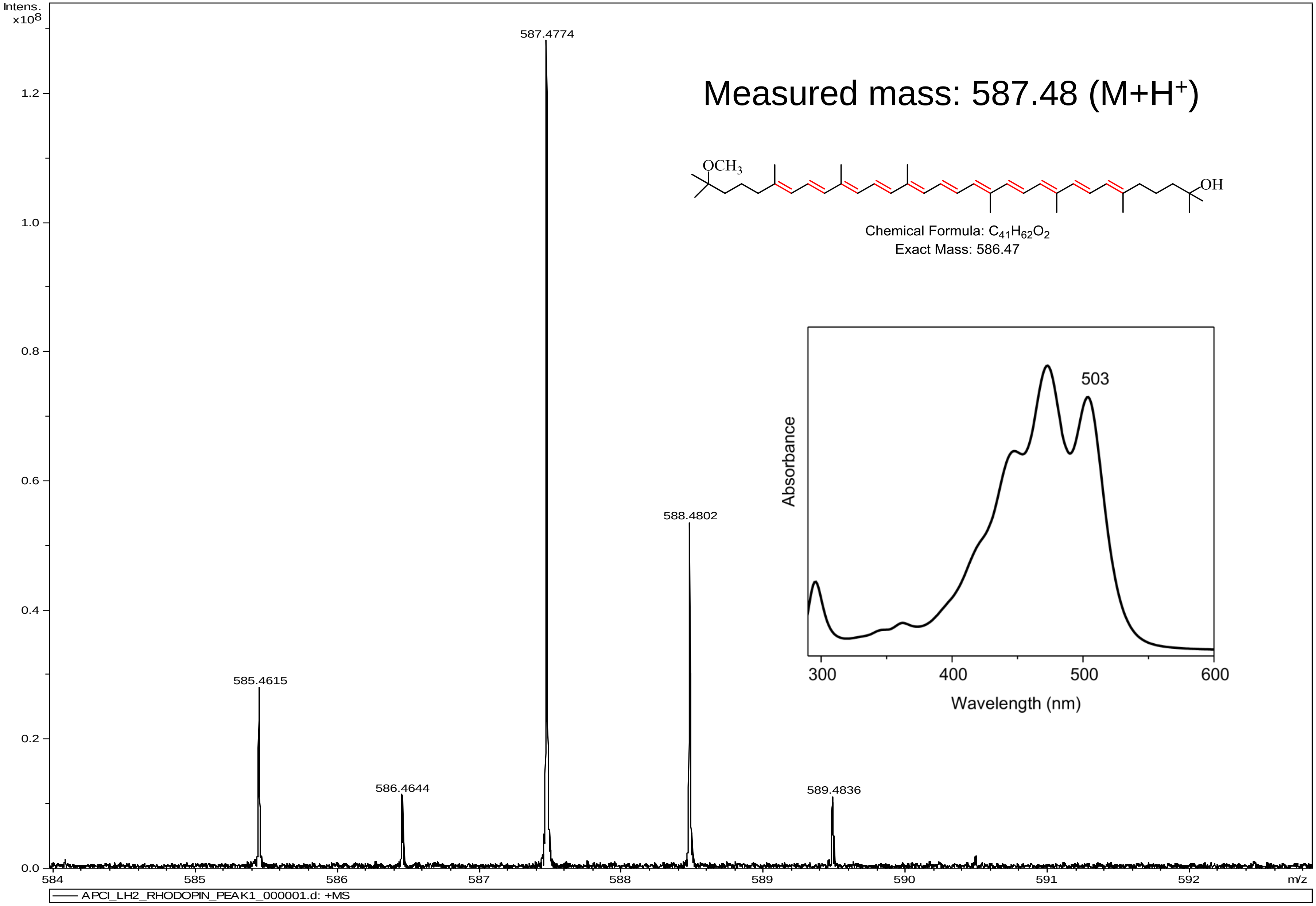

Peak 11: Anhydrorhodovibrin

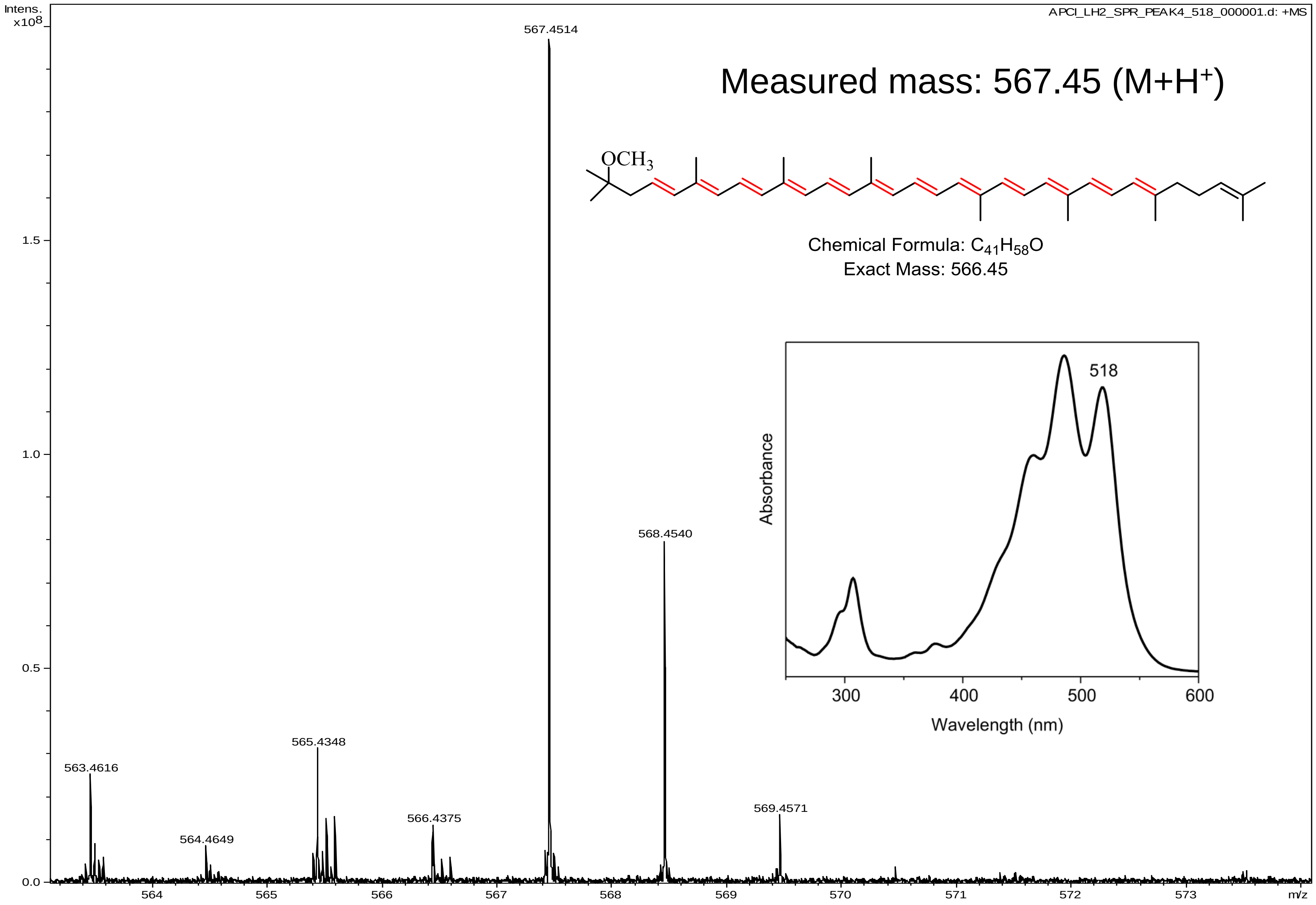

Peak 12: Spirilloxanthin

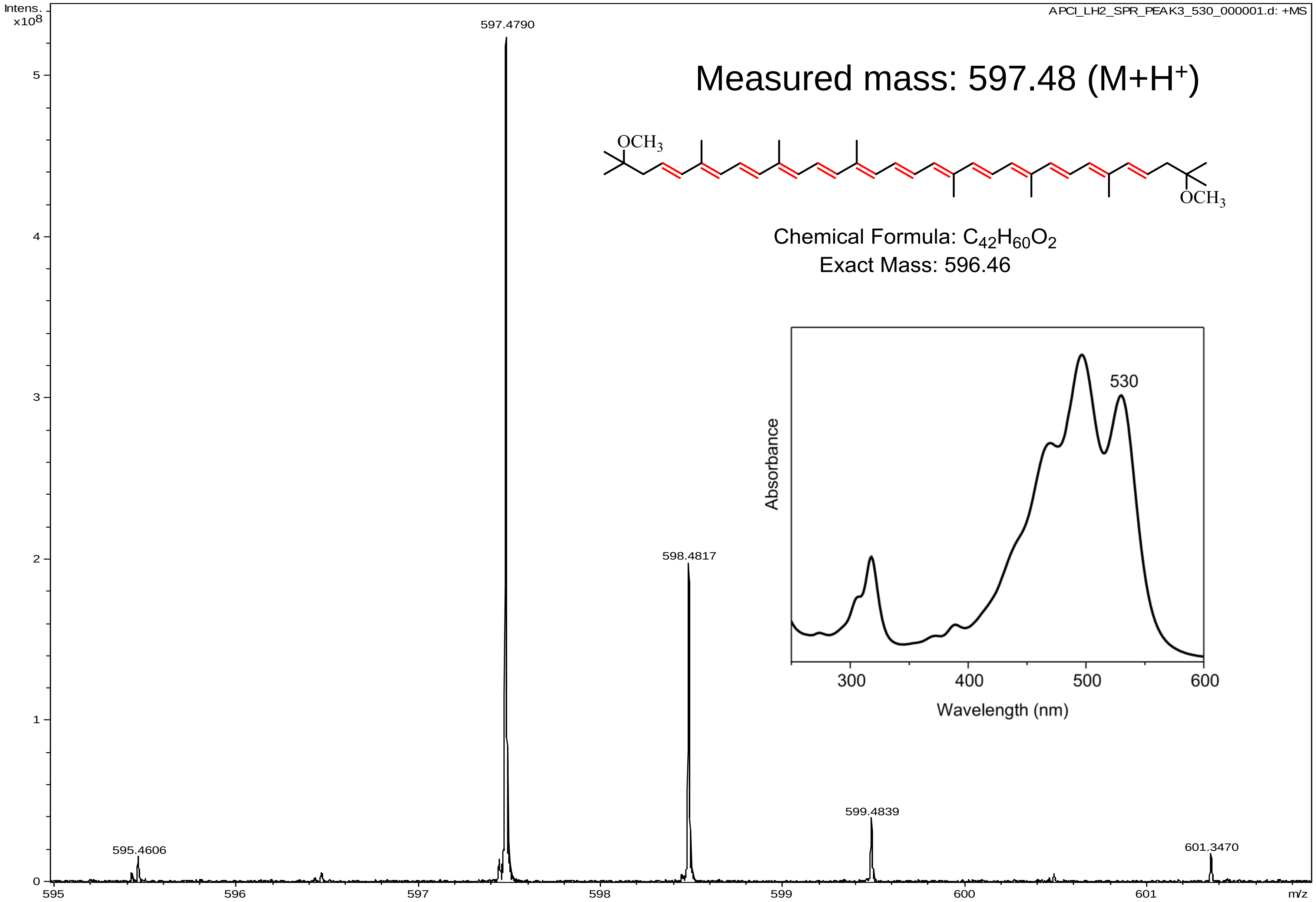

Peak 13: Di-dehydro-rhodopin

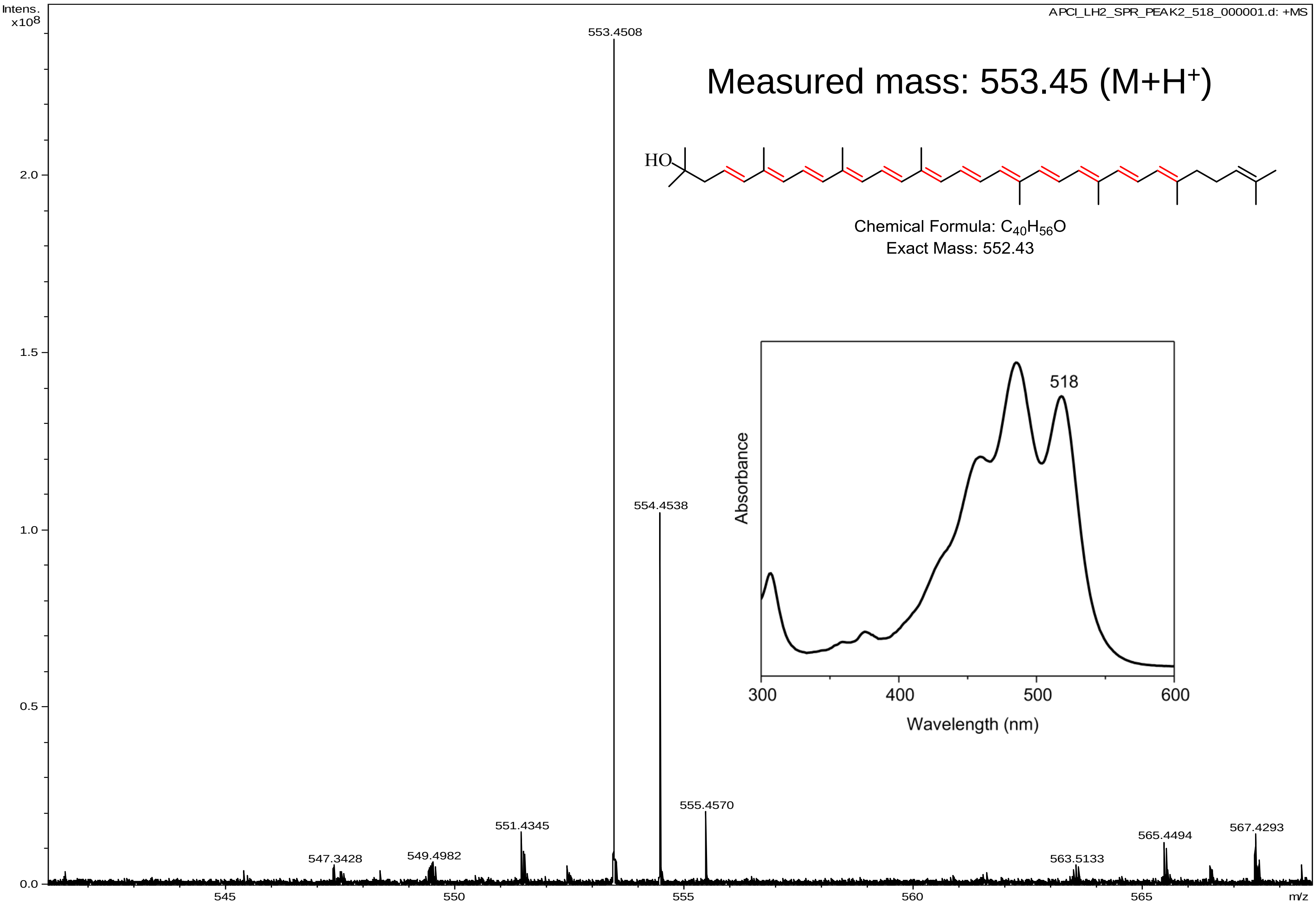

Peak 14: Keto-anhydrorhodovibrin

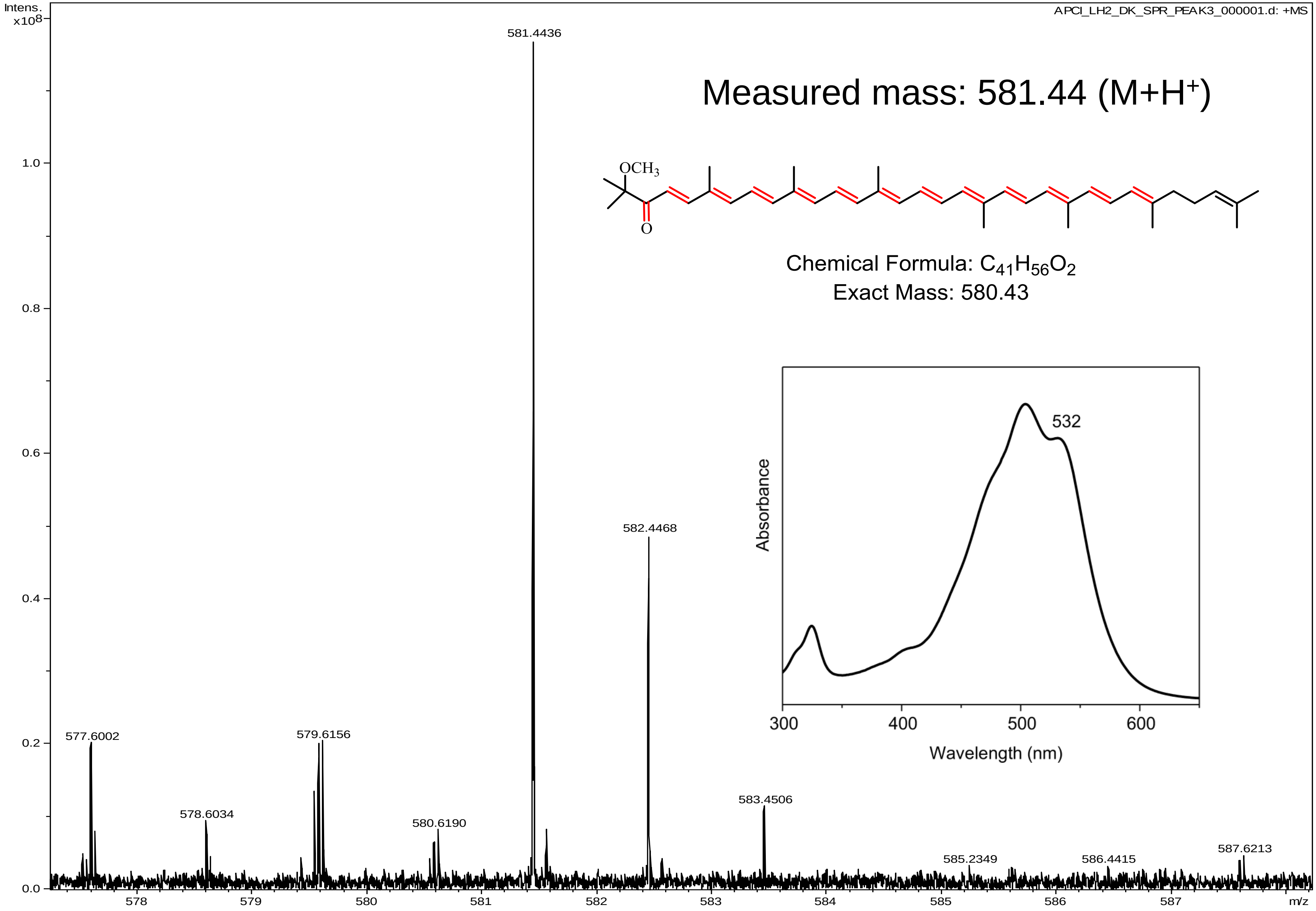

Peak 15: Keto-spirilloxanthin

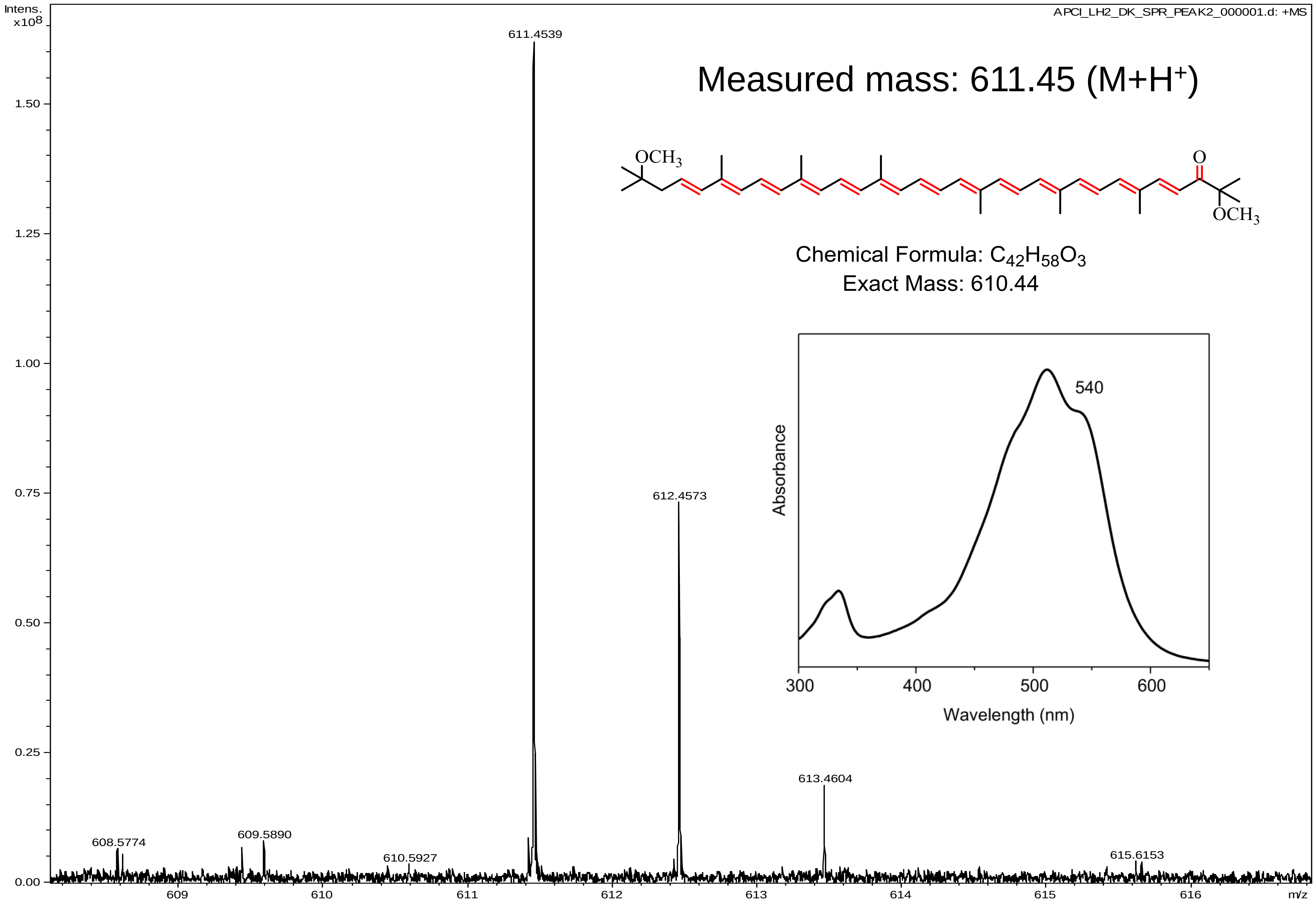

Peak 16: Di-keto-spirilloxanthin

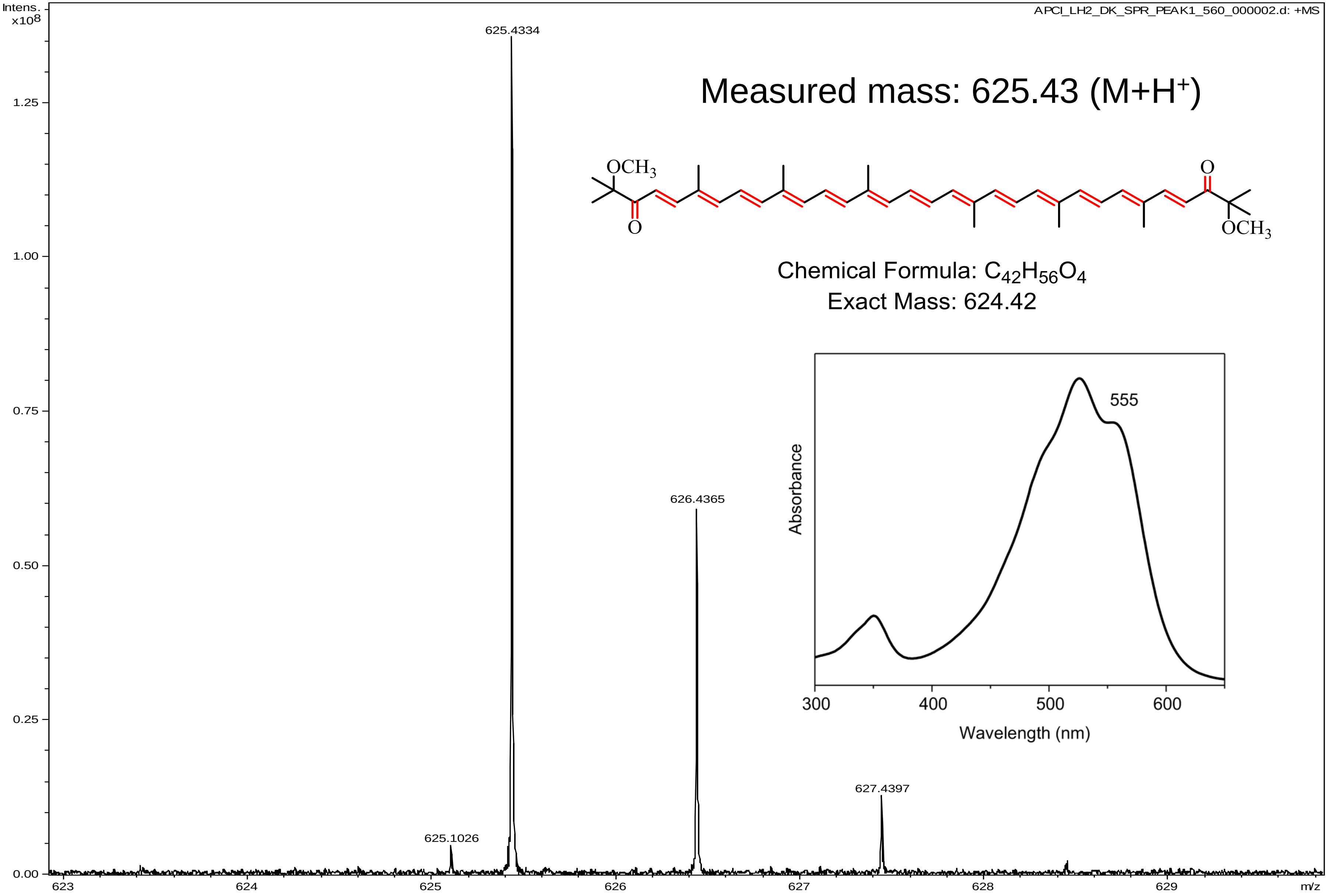

Supplement: Fig. S1 — Mass spectrometry of carotenoids corresponding to peaks 1, 3–16 in the HPLC analysis shown in Fig. 5. Each panel shows the MS analysis, the measured mass, structure, chemical formula and exact mass of the carotenoid. Each inset absorption spectrum is taken from the HPLC analysis. [file mmc1.pdf]
